# Cheminformatics-Based Identification of Potential Novel Anti-SARS-CoV-2 Natural Compounds of African Origin

**DOI:** 10.3390/molecules26020406

**Published:** 2021-01-14

**Authors:** Samuel K. Kwofie, Emmanuel Broni, Seth O. Asiedu, Gabriel B. Kwarko, Bismark Dankwa, Kweku S. Enninful, Elvis K. Tiburu, Michael D. Wilson

**Affiliations:** 1 Department of Biomedical Engineering, School of Engineering Sciences, College of Basic and Applied Sciences, University of Ghana, Legon P.O. Box LG 54, Accra, Ghana; skkwofie@ug.edu.gh (S.K.K.); ebroni002@st.ug.edu.gh (E.B.); etiburu@ug.edu.gh (E.K.T.); 2 West African Centre for Cell Biology of Infectious Pathogens, Department of Biochemistry, Cell and Molecular Biology, University of Ghana, Legon P.O. Box LG 54, Accra, Ghana; gabrielbrako@ymail.com; 3 Department of Medicine, Loyola University Medical Center, Maywood, IL 60153, USA; 4 Department of Parasitology, Noguchi Memorial Institute for Medical Research, University of Ghana, Legon P.O. Box LG 581, Accra, Ghana; sethasieduosei@gmail.com (S.O.A.); bdankwa@noguchi.ug.edu.gh (B.D.); kenninful@noguchi.ug.edu.gh (K.S.E.)

**Keywords:** SARS-CoV-2, coronavirus, African natural products, molecular docking, virtual screening, molecular dynamics, SARS-CoV-2 inhibitors

## Abstract

The coronavirus disease 2019 (COVID-19) pandemic caused by the severe acute respiratory syndrome virus 2 (SARS-CoV-2) has impacted negatively on public health and socioeconomic status, globally. Although, there are currently no specific drugs approved, several existing drugs are being repurposed, but their successful outcomes are not guaranteed. Therefore, the search for novel therapeutics remains a priority. We screened for inhibitors of the SARS-CoV-2 main protease and the receptor-binding domain of the spike protein from an integrated library of African natural products, compounds generated from machine learning studies and antiviral drugs using AutoDock Vina. The binding mechanisms between the compounds and the proteins were characterized using LigPlot+ and molecular dynamics simulations techniques. The biological activities of the hit compounds were also predicted using a Bayesian-based approach. Six potential bioactive molecules NANPDB2245, NANPDB2403, fusidic acid, ZINC000095486008, ZINC0000556656943 and ZINC001645993538 were identified, all of which had plausible binding mechanisms with both viral receptors. Molecular dynamics simulations, including molecular mechanics Poisson-Boltzmann surface area (MM/PBSA) computations revealed stable protein-ligand complexes with all the compounds having acceptable free binding energies <−15 kJ/mol with each receptor. NANPDB2245, NANPDB2403 and ZINC000095486008 were predicted as antivirals; ZINC000095486008 as a membrane permeability inhibitor; NANPDB2403 as a cell adhesion inhibitor and RNA-directed RNA polymerase inhibitor; and NANPDB2245 as a membrane integrity antagonist. Therefore, they have the potential to inhibit viral entry and replication. These drug-like molecules were predicted to possess attractive pharmacological profiles with negligible toxicity. Novel critical residues identified for both targets could aid in a better understanding of the binding mechanisms and design of fragment-based *de novo* inhibitors. The compounds are proposed as worthy of further in vitro assaying and as scaffolds for the development of novel SARS-CoV-2 therapeutic molecules.

## 1. Introduction

Coronavirus disease 2019 (COVID-19) is caused by severe acute respiratory syndrome coronavirus 2 (SARS-CoV-2) [1,2]. As of 8 December 2020, the novel coronavirus disease 2019 (COVID-19) has spread globally, with 66,729,375 confirmed cases, including 1,535,982 deaths [3]. The disease outbreak, declared a pandemic, has led to nearly three billion people in about 82 countries under partial or full lockdowns due to the infections [4]. The mild symptoms include fever, dry cough, runny nose, sore throat, and difficulty in breathing [5]. Chemosensory dysfunctions such as ageusia (loss of taste) and anosmia (loss of smell) have also been reported [6,7,8,9]. In more severe cases, the symptoms include severe muscle pain, cardiovascular shock, arrhythmia, acute respiratory distress syndrome (ARDS), and hyper inflammation [5,10]. The infection is spread mainly from one person to others via droplets produced from the respiratory systems of infected people, often during coughing or sneezing [5].

SARS-CoV-2, a positive-sense single-stranded RNA virus, is a member of the coronaviridae family, which are enveloped nonsegmented viruses with large surface spike proteins [11]. Most of the RNA encodes for RNA synthesis material, viral polymerase (RdRp), and nonstructural polyproteins [12,13], while the remainder encodes four structural proteins comprising spike (S), envelope (E), membrane (M), nucleocapsid (N), and other helper proteins [12]. 

There is evidence that the SARS-CoV-2 infects the host’s cells that co-express both angiotensin-converting enzyme 2 (ACE2) and transmembrane protease serine 2 (TMPRSS2). The ACE2 binds to the spike protein, facilitating the viral cell entry, and TMPRSS2 proteolytically cleaves it, resulting in fragments that activate cell–cell and virus–cell fusion and interferes with antibody-mediated neutralization [14]. This suggests that TMPRSS2 might impact the spread of the virus via two independent mechanisms [14]. The S1 subunit of the spike protein binds to the ACE2 receptor, and the S2 subunit is responsible for the fusion between the virion and host receptors [15,16]. Upon binding to ACE2, the virion releases its RNA into the cell and takes over the host cell machinery to produce copies of itself, which are then shed via exocytosis to infect fresh cells [12,17,18,19].

The viral main protease (M^pro^) and the papain-like proteases are critical in the translation of viral RNA to polyproteins. The viral spike protein also possesses a variable receptor-binding domain (RBD), which, if inhibited, should prevent viral attachment, fusion and entry, and thus make it also another attractive target [15]. 

Although, there are no FDA-approved drugs for the treatment and prevention of COVID-19, several antiviral compounds including remdesivir, favipiravir and darunavir; anti-HCV; nucleotide inhibitors sofosbuvir, IDX-184 and ribavirin; and kinase inhibitor imatinib are being repurposed [20,21,22,23,24]. Some of these repurposed drugs are currently undergoing various phases of clinical trials. Others such as dexamethasone and remdesivir have been given provisional approval for COVID-19 treatment [25,26]. The outcomes of these efforts are not assured, therefore emphasizing the urgent need to identify novel molecules that are therapeutically efficacious and maximally safe to human hosts.

Considering the urgency that the current situation of the pandemic demands, the conventional approach to drug discovery is time-consuming, but computational methods offer cost-effective and faster alternatives of discovering novel compounds. A network-based approach was used to predict repurposed drugs or combination therapies for SARS-CoV-2 by quantifying the interplay between drugs and human coronaviruses (HCoVs)–host interactome [27]. Additionally, a total of 1000 compounds were prioritized for downstream analysis as potential inhibitors via screening using the deep docking of 1.3 billion compounds present in the ZINC15 library against SARS-CoV-2 M^pro^ [28]. Camostat mesylate, which is an inhibitor of serine protease TMPRSS2, was shown to block cell entry of SARS-CoV-2 [17]. Other efforts geared towards the unraveling of potential antivirals targeting SARS-CoV-2 have been reported [24,29,30,31,32]. Nevertheless, issues of therapeutic potency persist, since potential promising molecules produced disappointing results during trials [33]. It is exigent to identify novel molecules that can disrupt critical mechanisms and biomolecular pathways involving SARS-CoV-2. These molecules must be therapeutically efficacious and safe to human hosts with negligible side effects.

Pharmacoinformatics methods have been applied in the development of antivirals such as Boceprevir, Saquinavir and Rupintrivir [34]. The viral M^pro^, alongside the papain-like proteases, is critical in the translation of viral RNA to polyproteins. The M^pro^ has been suggested as a drug target, and its inhibition can obstruct viral replication [35,36]. Furthermore, the cleavage specificity of the main protease is absent in human proteases, suggesting potential inhibitors are unlikely to be toxic to the human host [36]. The enzyme spike protein is also another attractive drug target [1], which plays an essential role in facilitating viral entry into target cells [17]. The spike proteins contain a variable receptor-binding domain (RBD) located in the S1 subunit, which binds to the ACE2 receptor found most abundantly in the lungs and organs, including the heart, kidneys, and gastrointestinal tract. The spike protein S2 subunit is responsible for the fusion between viral and host receptors [15,16]. Inhibiting the viral spike protein will occlude viral attachment, fusion and entry [15]. 

The structural and chemical diversity of natural product-derived compounds serve as rich sources of scaffolds for the discovery of novel drug leads [37,38]. Natural products have traditionally played a prominent role in treatment [39]. Currently, natural products and their derivatives represent over one-third of all new molecular entities (NMEs) approved by the US FDA [40]. Cheminformatics databases, including the Traditional Chinese Database [41], African Natural Product Database (AfroDB) [42], and North African Natural Product Database [43], collectively contain over 30,000 freely accessible unique natural product compounds. Natural products including saikosaponins and raoulic acid have been reported to exhibit antiviral properties against coronaviruses [44].

The recent strides in understanding the genome of SARS-CoV-2 and the available plethora of diverse structural genomic data combined with advanced high-performance biocomputing are key in expediting the identification of COVID-19 drugs. Therefore, this study sought to identify promising novel molecules with the potential of disrupting the critical mechanisms involving the SARS-CoV-2, including viral attachment, entry and replication processes using cheminformatics. The study seeks to virtually screen an integrated library made up of diverse African natural compounds [45] and recently prioritized hits from a deep docking study of 1.3 billion compounds [28] together with drugs currently undergoing clinical trials against SARS-CoV-2 M^pro^ and RBD of the spike protein. This is to identify potential polypharmacological antiviral compounds targeting both proteins [46,47] with novel scaffolds to augment the design of next-generation inhibitors against the cell entry and replication of SARS-CoV-2. Moreover, we sought to gain novel insights into the mechanisms of binding between the receptors and compounds using molecular dynamics (MD) simulations, including molecular mechanics Poisson-Boltzmann surface area (MM/PBSA) methods, by evaluating their binding free energies [48]. A Bayesian-based technique was also used to predict the antiviral activity of the compounds as a measure to characterize their anti-SARS-CoV-2 propensities. 

## 2. Results and Discussion

### 2.1. Description of Binding Sites of M^pro^ and RBD Structures

**Figure 1:**
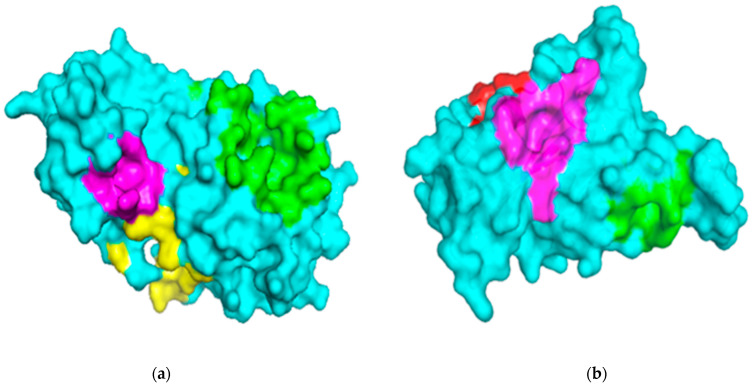
Surface representations of the protein structures generated using PyMOL. (**a**) The structure of severe acute respiratory syndrome virus 2 main protease (SARS-CoV-2 M^pro^) is colored cyan, and the predicted binding pockets 1, 2, and 3 are colored green, yellow and magenta, respectively. (**b**) The receptor-binding domain (RBD) of the spike protein is colored cyan, and the predicted binding pockets A, B, and C are colored green, red and magenta, respectively.

The binding sites of the M^pro^ and RBD of the spike protein structures were characterized using the Computed Atlas of Surface Topography of proteins (CASTp) version 3.0 (available at http://sts.bioe.uic.edu/castp/calculation.html). CASTp utilizes theoretical and algorithmic results of computational geometry to analytically predict pockets and cavities while excluding shallow depressions from the calculations [49]. The predicted binding cavities were analyzed using Chimera version 1.12 and PyMOL. The volumes and areas of the sites were also determined using Chimera version 1.12 (Table 1). Predicted binding sites with very small volumes and areas such that no ligands could fit were not considered for downstream virtual screening. The surface representations of the two proteins with three putative binding pockets are shown (Figure 1). 

#### 2.1.1. Binding Site Analysis of M^pro^

The three-dimensional structure of the M^pro^ SARS-CoV-2 solved using X-ray diffraction at a resolution of 1.31 Å is a homodimer with a sequence length of 306 along one chain and shares a 96% sequence identity to the SARS-CoV M^pro^ [36]. The plausible binding sites of the SARS-CoV-2 M^pro^ were predicted via CASTp (Table 1) using the M^pro^ structure with a Protein Data Bank (PDB) ID 5R82. 

Ser1, His41, Met49, Gly143, Phe140, Ser144, Cys145, His163, His164, Glu166, Pro168 and Gln189 have been determined in previous studies as lining the active sites of M^pro^ [36,50], consistent with the prediction by CASTp as Pocket 1 (Table 1). A computational study to identify potential SARS-CoV-2 M^pro^ inhibitors reported that the top compound docked into the M^pro^-binding cavity lined by residues His41, Met49, Tyr54, Phe140, Leu141, Asn142, Ser144, Cys145, His163, Met165, Glu166, Leu167, Pro168, Asp187, Arg188, Gln189 and Gln192 [28]. Other studies have also reported that residues of M^pro^ forming hydrogen bonds include Gly143, His163, His164, Glu166, Gln189 and Thr190, with Cys145 forming a covalent bond [29].

Additionally, the region around Glu288-Asp289-Glu290 has been reported to be involved in a likely enzyme dimerization in SARS-CoV [51]. Mutating Glu288, Asp289, Glu290, Arg298 and Gln299 led to a high decrease in enzymatic activities of the protease, and this region overlaps with pockets 2 and 3 (Table 1). Additionally, mutations in Asn214, Leu282 and Cys300 significantly decreased the activity of the protease. Furthermore, the replacement of Ser284, Thr285, Ile286 or Phe291 by Ala produced mutated proteases with higher enzymatic activities than the wild type [51]. Leu282, Ser284 and Phe291 were predicted as residues lining pocket 3 (Table 1).

A molecular docking study of compounds against M^pro^ identified possible binding sites of the protein. Remdesivir was reported to dock in the region lined by residues Gln107, Pro108, Pro132, Ile200, Glu240 and His246 [52]. This binding site was also identified by other computational studies [53,54]. Herein, these residues were predicted as lining pocket 4 (Table 1). Pockets 1, 2, 3, 4 and 5 were selected as the most plausible binding cavities for the main protease (Table 1) and were considered for the virtual screening process.

#### 2.1.2. Binding Site Analysis of RBD

The 3D structure of the RBD of the spike protein was obtained from the PDB database with ID 6M0J [55]. The RBD was solved using X-ray diffraction at a resolution of 2.45 Å. The receptor-binding domain is located on chain E of the spike protein with a residue count of 229 [55]. A previous study identified a significant difference between the C-terminus residues of the RBDs of SARS-CoV and SARS-CoV-2, although this did not affect the capability to engage ACE2 [56]. The binding sites of both RBD proteins of SARS-CoV-2 and SARS-CoV are highly conserved [57]. 

Lys417, Gly446, Tyr449, Tyr453, Leu455, Phe456, Phe486, Asn487, Tyr489, Gln493, Gly496, Gln498, Thr500, Asn501, Gly502 and Tyr505 are contacting residues of the RBD at the SARS-CoV-2 RBD–ACE2 interface [55]. An in silico study to repurpose FDA-approved drugs as SARS-CoV-2 spike protein inhibitors via virtual screening revealed the binding of compounds occurred at the SARS-CoV-2 RBD–ACE2 interface [58]. 

In a recent computational study to identify anti-SARS-CoV-2 spike protein molecules, the compounds were reported to interact with at least one of the following residues: Leu335, Cys336, Phe337, Phe338, Gly339, Val341, Phe342, Asn343, Ala344, Thr345, Lys356, Asp364, Val367, Leu368, Tyr369, Asn370, Ser373, Phe374, Phe377, Lys378, Cys379, Ala397, Asn422, Gly431, Trp436, Leu441, Arg509, Val510, Val511, Phe515 and Asn643 [59]. Cys336 is a critical residue for the RBD, since it forms a disulfide bond with Cys361, which helps stabilize the β sheet structure [55]. A NAG glycan has also been reported to be linked to Asn343 of the RBD [55], which was identified as a critical residue in our study. These residues overlap with pockets B and C, as predicted (Table 1). For the RBD, pockets A, B and C (Table 1) and the RBD–ACE2 interface were considered as plausible binding sites.

### 2.2. Virtual Screening Studies

#### 2.2.1. Molecular Docking Studies

Molecular docking is an essential technique in computer-aided drug discovery [60]. With the structure of the receptor known, compounds of interest are screened in silico to guide the selection of potential leads. AutoDock Vina used for docking employs an empirical and knowledge-based scoring function to predict the binding affinity of compounds [61]. The grid boxes for both receptors were set to cover all the binding sites (Table 1). Compounds with binding energies of −7.5 kcal/mol or less for both receptors were selected for the downstream analysis. This threshold was used, since −7.0 kcal/mol has been reported to significantly discriminate between putative specific and nonspecific protein–ligands for viruses [62]. A total of 1462 ligands from the African natural compounds (ANC) library were successfully screened against both the M^pro^ and RBD, whilst 940 compounds from the ML study were screened against both receptors, including 43 known antivirals. 

##### Molecular Docking Studies of M^pro^

A grid box with a dimension of 37.58 * 64.78 * 62.77 Å^3^ and center 49.33, 49.36 and 49.56 Å was specified for M^pro^. Out of 1462 successfully screened ANC compounds, 65 had a binding affinity ≤−7.5 kcal/mol. A total of 112 compounds from the ML library and 14 known drugs met the threshold. Ledipasvir had the highest binding affinity of −9.6 kcal/mol to the M^pro^ (Table S1). Velpatasvir and imatinib also had binding affinities of −8.9 and −8.5 kcal/mol, respectively (Table S1). Other studies have proposed ledipasvir and velpatasvir as potential anti-SARS-CoV-2 M^pro^ molecules [63,64]. After using AutoDock to screen FDA-approved drugs against the M^pro^, velpatasvir was reported to possess a binding affinity of −9.1 kcal/mol [63]. Imatinib, currently in phase 3 clinical trials for adults hospitalized with SARS-CoV-2 (https://clinicaltrials.gov/ct2/show/record/NCT04394416), was also reported to demonstrate the highest binding affinity (−11.46 kcal/mol) to the M^pro^ in a recent study [65]. These results are consistent with the docking outcomes reported herein. The differences in binding affinity values could be due to the various software packages used and the different preparation protocols the protein and ligand structures were subjected to.

For the ANC library, ZINC000095486008, NANPDB2403 (retusolide B), NANPDB2245 (helioscopinolide B) and NANPDB2510 (jolkinolide E) demonstrated binding affinities of −8.2, −8.1, −8.0 and −7.9 kcal/mol, respectively, with the M^pro^ (Table 2). ZINC001657931232 had the highest binding affinity of −8.4 kcal/mol to the M^pro^ among the ML compounds. ZINC001181689720 and ZINC001460974086 also had binding affinities of −8.3 and −8.2 kcal/mol, respectively (Table S1). Remdesivir, hydroxychloroquine and chloroquine had low binding affinities of −6.8, −5.9 and −5.5 kcal/mol with the M^pro^, respectively (Table 2). In the quest of finding potential SARS-CoV-2 inhibitors, antiviral and antimalarial drugs were virtually screened against the M^pro^ (PDB ID: 6LU7) and S-protein. Remdesivir, hydroxychloroquine, and chloroquine were reported to possess binding affinities of −6.5, −5.3 and −5.1 kcal/mol with the M^pro^, respectively, consistent with the results obtained herein [66]. Although, the binding affinities are relatively low, it does not exclude them as anti-SARS-CoV-2 M^pro^ molecules.

##### Molecular Docking Studies of RBD

For the RBD, the grid box was set with dimensions of 42.54 * 42.73 * 42.44 Å^3^ and centered at 41.19, 47.74, and 55.37 Å. A total of 26 compounds from the ANC library, 22 from the ML-based study and 9 known drugs demonstrated binding affinities ≤−7.5 kcal/mol, thereby complying with the threshold. Ledipasvir showed the highest binding affinity (−9.9 kcal/mol), followed by velpatasvir and imatinib, which also had binding affinities of −8.5 and −8.1 kcal/mol, respectively (Table S1). A recent study reported that ledipasvir and velpatasvir have high binding affinities of −8.4 and −7.9 kcal/mol with the spike glycoprotein, respectively [67]. 

From the ANC library, ZINC000095486008, NANPDB2403 (retusolide B), NANPDB2245 (helioscopinolide B) and NANPDB2510 (jolkinolide E) had binding affinities of −7.8, −7.8, −7.7 and −7.6 kcal/mol, respectively (Table 2). For the ML library, ZINC001657931232, ZINC001181689720 and ZINC001460974086 had the highest binding affinities to the M^pro^ with binding affinities of −7.8, −7.5 and −7.6 kcal/mol, respectively. Remdesivir, hydroxychloroquine and chloroquine also had low binding affinities of −6.3, −5.5 and −4.9 kcal/mol, respectively (Table 2).

##### Shortlisted Compounds for Downstream Analysis

Thirteen compounds from the ANC library, fourteen from the ML library and nine known antivirals met the threshold for both RBD and M^pro^ (Table 2). In total, 36 compounds, including experimental drugs had binding affinities of ≤−7.5 kcal/mol with both receptors (Table 2) and were shortlisted for further analysis.

#### 2.2.2. Characterization of the Protein–Ligand Interactions

The nature of the active site and the functional groups on the ligands are critical for stabilization within the binding pocket of a receptor [68]. Studies into these interactions are key in determining whether a ligand is considered as a promising lead. The protein-ligand interactions of the 36 shortlisted compounds were studied using LigPlot+ [69] and PyMOL. After analyzing their binding interactions, five compounds comprising NANPDB2403, NANPDB2245, ZINC000055656943, ZINC000095486008 and ZINC001645993538 were selected as hits (Table 2). The interactions of the known antivirals and experimental drugs with the respective targets were compared. Characterizing the binding interactions enabled the identification of certain critical residues within the active pockets of the respective protein targets. 

##### Characterization of the M^pro^–Ligand Interactions

**Figure 2:**
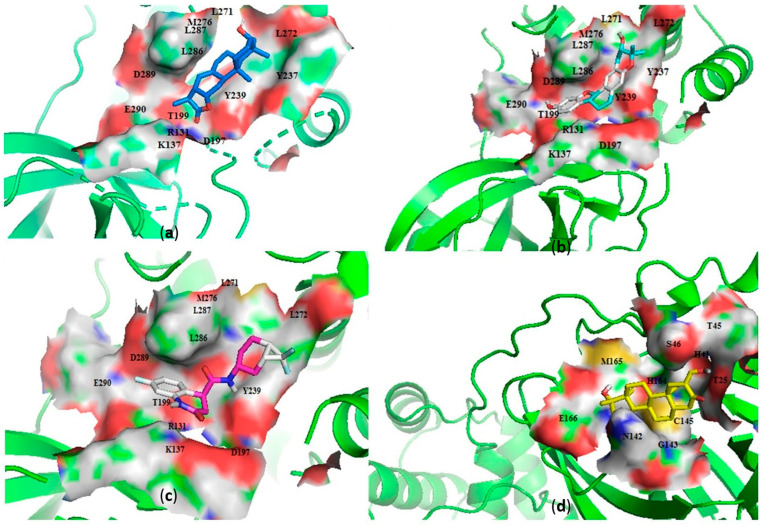
Cartoon representation of the main protease (M^pro^) in complex with: (**a**) NANPDB2245 (helioscopinolide B), (**b**) ZINC000095486008, (**c**) ZINC001645993538, and (**d**) oxymetholone. The binding sites are shown as a surface representations, with the ligands shown as sticks.

Considering the molecular interactions of the M^pro^, all the 36 compounds that met the ≤−7.5 kcal/mol threshold, except for ZINC000544552417 and ZINC000621286015, formed at least one hydrogen bonding with the M^pro^ residues. Oxymetholone docked into pocket 1 (Figure 2d, and Table 1 and Table 2), the known active site of the M^pro^ [36,50]. It formed a hydrogen bond with Thr25 (bond length 2.81 Å); two with Glu166 (bond lengths 2.9 Å and 3.00 Å); and hydrophobic bonds with His41, Ser46, Thr45, Asn142, Gly143, Cys145, His164 and Met165. A recent study also revealed that tipranavir, which is a nonpeptidic protease inhibitor used in combination with ritonavir to treat HIV, interacted with Gln192 and Met165 (both formed hydrogen bonds) and Gln189, Asp187, Met49, Arg188, Ser46, Cys44, Thr25 and His41 in a different conformation from that of the α-ketoamide inhibitor [70]. It was further identified that raltegravir demonstrated a high binding affinity to the M^pro^ than the co-crystallized α-ketoamide, with interactions from His164, Arg188, Gln192, Glu166, Met49, Met165, Phe140, Pro168 and Leu167 [70]. Considering our results and references to existing literature, we suggest that Gly143, Cys145 and Glu166 are critical residues for binding.

**Figure 3:**
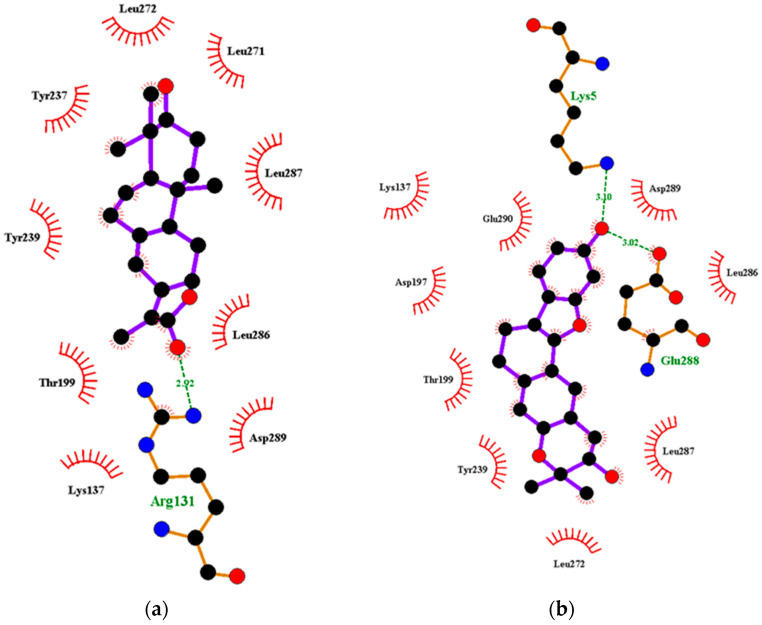
Two-dimensional diagrams of the M^pro^–ligand interactions generated using LigPlot+. (**a**) Interaction profiles of the M^pro^–NANPDB2245, and (**b**) M^pro^–ZINC000095486008 complexes. Ligands are colored in purple, hydrogen bonds are represented as green dash lines, and hydrophobic contacts are represented as red spoke arcs.

Interestingly, ledipasvir, NANPDB2403, NANPDB2245 (Figure 3a), ZINC0095486008 (Figure 3b), and ZINC001645993538 (Figure 2c) were found to interact with the main protease in a different binding cavity with binding energies of −9.6, −8.1 and −8.0, respectively (Table 2). The residues lining this cavity include Arg131, Lys137, Thr199, Tyr237, Tyr239, Leu271, Leu272, Gly275, Leu286, Leu287, Glu288, Asp289 and Glu290. This binding pocket is located between pockets 2, 3 and 4. Other in silico studies also identified this binding cavity [71,72]. Ledipasvir formed hydrogen bonding with Met276 of a bond length 2.92 Å and hydrophobic contacts with Lys5, Gly124, Tyr126, Gln127, Lys137, Gly138, Ser139, Thr199, Tyr237, Tyr239, Leu272, Gly275, Asn277, Gly278, Leu286, Leu287 and Glu290. NANPDB2245 formed a hydrogen bond with Arg131 (bond length 2.92 Å) and nine hydrophobic contacts with M^pro^ residues comprising Lys137, Thr199, Tyr237, Tyr239, Leu271, Leu272, Leu286, Leu287 and Asp289 (Table 2 and Figure 2a). ZINC000095486008 formed two hydrogen bonds with Lys5 (bond length 3.1 Å) and Glu288 (bond length 3.02 Å), and interacted with Lys137, Asp197, Thr199, Tyr239, Leu272, Leu286, Leu287, Asp289, and Glu290 via hydrophobic contacts (Table 2 and Figure 2b). 

##### Characterization of the RBD–Ligand Interactions

A total of 27 out of the 36 compounds formed hydrogen bonds of varying lengths with the RBD (Figure S1 and Table S1). Ledipasvir interacted via hydrogen bonding with Gly339 and formed hydrophobic contacts with Leu335, Cys336, Pro337, Phe338, Phe342, Asn343, Ala363, Asp364, Leu368, Ser371, Ala372, Ser373, Phe374, Ser375, Trp436, Asn437 and Tyr508 (Table S1). 

Oxymetholone formed hydrogen bonding with Cys336 (bond length of 3.0 Å) and Asn343 (bond length of 3.09 Å); and hydrophobic contacts with Leu335, Phe338, Gly339, Phe342, Asp364, Val367, Leu368, Ser371 and Phe374 (Table 2). ZINC000095486008 also interacted with the RBD via three hydrogen bonds with Cys336, Phe338 and Gly339 of bond lengths 2.96, 3.26 and 3.3 Å, respectively (Table 2 and Figure S3B). NANPDB2245 interacted via nine hydrophobic contacts Leu335, Cys336, Phe338, Gly339, Asp364, Val367, Leu368, Ser371 and Phe374, and formed one hydrogen bond with Asn343 (Figure S3A). Therefore, we suggest Cys336, Ser373 and Phe374 as potential critical residues. 

The residues Leu455, Phe486 and Gln493 of RBD have been reported to interact with Lys31 (hotspot 31), whereas residues Asn487 and Ser494 of the RBD are described to interact with Lys353 (hotspot 353) of the human ACE2 [73,74]. Compounds KT185, KT203, GSK1838705A, BMS195614 and RS504393 were reported to bind to the RBD by interacting with Leu455, Phe486, Asn487, Gln493 and Ser494 [75]. These compounds were predicted to interact and block key RBD residues responsible for recognizing hotspot 31 and hotspot 353 of SARS-CoV-2 [75].

#### 2.2.3. Predictions of Biological Activities 

The biological activities for the 36 shortlisted compounds were elucidated. All the 13 ANC compounds shortlisted and two of the machine learning compounds were predicted to be antivirals, with a probable activity (Pa) > 0.3 and Pa > Pi (probable inactivity). Furthermore, the propensity of the compounds to be cell adhesion molecule inhibitors and membrane permeability inhibitors was considered in this study. The mechanisms of entry into the host cell are key in the survival of viruses, including coronaviruses [76]. More so, the first step in coronavirus entry is the adhesion to the host cell surface. The inhibition of SARS-CoV-2 spike proteins is a critical antiviral strategy, since it serves as the major barrier to block the infection [15,77]. A total of seven ANC and one machine learning compound were predicted to be cell adhesion inhibitors or membrane permeability inhibitors. The stimulation of human nuclear factor kappa B (NF-κB) transcription factors has been linked to the mediation of the induction of antiviral and inflammatory responses [78]. NANPDB2403 was predicted to be a cell adhesion inhibitor with a Pa of 0.760 and Pi of 0.005; anti-influenza activity with a Pa of 0.543 and Pi of 0.021; an anti-rhinovirus with a Pa of 0.499 and Pi of 0.02; and an anti-herpes activity with a Pa of 0.366 and Pi of 0.068. NANPDB2245 was predicted to possess anti-herpes activity with a Pa of 0.446 and Pi of 0.019. The ZINC000095486008 was also predicted to have anti-rhinovirus activities with a Pa of 0.511 and Pi of 0.021; an anti-influenza with a Pa of 0.431 and Pi of 0.037; and anti-herpes with a Pa of 0.431 and Pi of 0.037. ZINC000095486008 possesses potential anti-Ebola activity with a free binding energy of −114.650 kcal/mol against the VP24 protein target [45]. Since the Pa > Pi, these compounds can be considered as prospective antivirals, necessitating further experimental testing. 

### 2.3. Existing Drugs Proposed as Potential Frontline Treatment Options

After molecular docking, nine known antivirals and experimental drugs comprising ledipasvir, velpatasvir, imatinib, dactinomycin, dolutegravir, bictegravir, oxymetholone, raltegravir and sirolimus were predicted to have high binding affinities (≤−7.5 kcal/mol) to both M^pro^ and RBD of the spike glycoprotein (Table S1). A recent study also proposed the use of ledipasvir and velpatasvir for the treatment of SARS-CoV-2 [64]. Since all these drugs are FDA-approved, more attention must be focused on exploring their therapeutic potentials against SARS-CoV-2.

#### 2.3.1. Similarity Search of Hits

A structural similarity search of the hits was conducted via DrugBank. The search revealed that fusidic acid is structurally similar to NANPDB2245 and NANPDB2403, with similarity scores of 0.729 and 0.717, respectively. The structural similarity search also revealed that betulinic acid is similar to oxymetholone, with a score of 0.712. Oxymetholone used to treat HIV/AIDS wasting syndrome had binding energies of −7.8 and −7.7 kcal/mol against M^pro^ and RBD, respectively (Table 2). 

#### 2.3.2. Fusidic Acid and Betulinic Acid as Potential Anti-SARS-CoV-2 Compounds 

Fusidic acid and betulinic acid were virtually screened against both the M^pro^ and RBD. Fusidic acid had binding affinities of −6.9 and −7.2 kcal/mol against the M^pro^ and RBD, respectively. Betulinic acid also demonstrated good binding affinities of −7.7 and −7.4 kcal/mol with the M^pro^ and RBD, respectively. A recent in silico study proposed the use of betulinic acid for the treatment of SARS-CoV-2, since it had a good binding affinity to the main protease [79].

The interaction profiles between the receptors and these two compounds were also investigated. Fusidic acid formed hydrogen bonds with Lys137 (bond length of 2.8 Å), Leu271 (bond length of 2.94 Å) and Leu272 (bond length of 3.05 Å), and hydrophobic contacts with Arg131, Asp197, Thr199, Tyr239, Gly275, Met276, Leu286 and Asp289 (Table S1). For the RBD, fusidic acid formed hydrogen bonding with Ser371 (bond length of 2.7 Å) and Ser373 (bond length of 2.92 Å); and hydrophobic bonding with Leu335, Cys336, Gly339, Phe342, Asn343, Val367, Leu368, Phe374 and Trp436, which lined binding pocket B (Table 1 and Table S1). Fusidic acid is a natural product-derived bacteriostatic antibiotic classified under both approved and investigational drug categories in DrugBank. Fusidic acid suppresses bacterial growth and enhances the clearance of infections by the immune system by inhibiting translocation during the synthesis of protein. Fusidic acid was reported to inhibit the replication of feline infectious peritonitis virus (FIPV) in vitro by significantly reducing the viral titer [80]. Since fusidic acid is already an FDA-approved drug for humans, this offers the opportunity to further explore its therapeutic potential against SARS-CoV-2.

Additionally, betulinic acid formed a hydrogen bond with Asp289 of a bond length 2.79 Å and interacted with Arg131, Lys137, Asp197, Thr198, Thr199, Tyr237, Tyr239, Leu271, Leu272, Gly275, Met276, Leu286 and Leu287 via hydrophobic bonds. For the RBD, betulinic acid formed two hydrogen bonds with Phe515 (lengths 2.73 Å and 3.16 Å) and one with Thr430 (2.71 Å). Betulinic acid also interacted with the RBD via hydrophobic interactions with residues Pro426, Asp428, Phe429, Lys462, Pro463, Phe464 and Ser514. Betulinic acid was also reported to possess inhibitory activity against SARS-CoV M^pro^ with an IC_50_ value of 10 µM [81]. Based on modelization studies, the inhibition by betulinic acid was attributed to the multiple hydrogen bonds formed between betulinic acid and the M^pro^ [81]. Betulinic acid is an HIV-1 inhibitor with an EC_50_ value of 1.4 µM [82]. Other studies have also shown that derivatives of betulinic acid can interfere with HIV-1 virus entry in cells [83,84]. Since the S2 subunit of the spike protein of SARS-CoV and the glycoprotein 41 (Gp41) of HIV-1 are similar, blocking of the entry or fusion of the SARS-CoV viral particles to the human cell membrane was proposed as anti-SARS-CoV mechanisms [81].

### 2.4. Molecular Mechanics/Poisson-Boltzmann Surface Area (MM/PBSA) Calculations

Molecular dynamics (MD) simulations are performed after docking to assess the predicted binding modes of the top-ranking compounds as a filter in silico or to guide chemical synthesis for hit optimization [85]. At a quantitative level, simulation-based methods provide substantially more accurate estimates of ligand binding affinities (free energies) [86]. To understand the biophysical basis of recognition of inhibitors, MM/PBSA is employed for each system to identify stable MD trajectories, and the results are evaluated based on the total binding free energy of the ligand-receptor complex [48,87]. Binding free energy (ΔG_bind_) is used to quantify the affinity of a ligand to its target and is the free energy difference between the ligand-bound state (complex) and the corresponding unbound states of proteins and ligands. Assessing the ΔG_bind_ of a series of ligands against a particular target can unravel those ligands with higher binding affinities with the target. Thus, the ΔG_bind_ calculations are important to gain in-depth knowledge about the binding modes of the hits in drug design [88]. The various contributing energy terms were computed in this study (Table 3). Binding free energies between the hits and the respective targets were calculated after a 10 ns production MD run of the respective complexes using GROMACS. The binding free energies between fusidic acid, oxymetholone and remdesivir with the respective targets were also calculated. 

#### 2.4.1. MM/PBSA-Binding Free Energy Computational Analysis of M^pro^

Talampicillin was found to have a binding energy of −11.17 kcal/mol and MM/PBSA-binding free energy of −2.8 kcal/mol (−11.7152 kJ/mol) [89]. Talampicillin formed a network of bonds with residues His41, Met49, Gly143, Cys145, Met165, Glu166, Leu167, Pro168, Gln189 and Gln192 [89]. ZINC000015988935 also had binding energy of −12.39 kcal/mol and free binding energy of −4.62 kcal/mol (−19.33008 kJ/mol). It also showed a p-sulfur interaction with Met49; seven hydrogen bonds with the residues Arg188, Asp187, Gln189, Ser144, Cys145 and Glu166; an alkyl interaction with Met165; and a p-alkyl interaction with Cys145 [89].

Herein, the binding free energies of hits ranged from −17.097 to −61.090 kJ/mol (Table 3). Fusidic acid demonstrated the highest binding affinity with a binding free energy of −61.090 kJ/mol, while ZINC000055656943 had the highest binding free energy of −17.097 kJ/mol. NANPDB2245, NANPDB2403, ZINC000095486008, ZINC001645993538, remdesivir and oxymetholone had binding free energies of −56.223, −58.132, −47.058, −53.785, −58.356 and −44.724 kJ/mol, respectively, with M^pro^ (Table 3). 

#### 2.4.2. MM/PBSA-Binding Free Energy Computational Analysis of RBD

For the RBD, ZINC000095486008 demonstrated the highest affinity with a binding free energy of −65.174 kJ/mol. NANPDB2245 demonstrated the highest binding free energy (−22.142 kJ/mol) with the RBD. NANPDB2403, fusidic acid, ZINC000055656943, ZINC001645993538, remdesivir and oxymetholone also had binding free energies of −53.140, −55.858, −43.096, −61.778, −44.471 and −64.742 kJ/mol, respectively (Table 3). The low binding free energies exhibited by these compounds make them promising anti-SARS-CoV-2 molecules worthy of in vitro studies.

### 2.5. Other Contributing Energy Terms

The nonpolar component of the solvation free energy is estimated by the molecular solvent-accessible surface area (SASA) [90]. The SASA analysis measures the interaction between complexes and solvents. The M^pro^–ligand complexes had SASA energies ranging from −2.692 to −13.726 kJ/mol. M^pro^-Remdesivir demonstrated the lowest SASA energy of −13.726 kJ/mol, while M^pro^-ZINC000055656943 had a SASA energy of −2.692 kJ/mol. M^pro^-ZINC000095486008, M^pro^-fusidic acid, M^pro^-NANPDB2245, M^pro^-ZINC001645993538, M^pro^-NANPDB2403 and M^pro^-oxymetholone also had −12.692, −12.623, −10.829, −10.702, −9.939, and −8.485 kJ/mol, respectively. For the RBD–ligand complexes, the SASA energy values observed were between −3.930 and −15.298 kJ/mol (Table 3). ZINC000095486008 demonstrated the lowest SASA energy value of −15.298 kJ/mol.

The energy terms van der Waals, electrostatic and polar solvation energies are useful for analyzing free binding energies. Studies have shown that electrostatic and van der Waals forces contribute predominantly and continuously to the binding energy, along with simulations that favor the binding of complexes [91,92]. All the M^pro^–ligand complexes demonstrated very low van der Waals energies ranging from −18.966 to −114.276 kJ/mol (Table 3). M^pro^-Remdesivir demonstrated the lowest van der Waals energy of −114.276 kJ/mol, while ZINC000055656943 showed the highest van der Waals energy of −18.966 kJ/mol (Table 3). Fusidic acid, ZINC000095486008, NANPDB2245, ZINC001645993538, NANPDB2403 and oxymetholone demonstrated van der Waals energies of −99.476, −98.620, −85.615, −84.952, −77.965 and −60.820 kJ/mol, respectively (Table 3). For the RBD–ligand complexes, the van der Waals energies ranged between −30.310 and −119.217 kJ/mol. RBD-ZINC000095486008 demonstrated the lowest van der Waals energy of −119.217 kJ/mol, while RBD-NANPDB2245 had −30.310 kJ/mol (Table 3).

Compounds with low electrostatic energies and high polar energies have been reported to be active against receptors [93]. The electrostatic energies ranged between −2.907 to −20.464 kJ/mol for the M^pro^–ligand complexes and −2.337 to −16.338 kJ/mol for the RBD–ligand complexes. (Table 3). High polar solvation energies were also observed for all protein–ligand complexes. For the M^pro^–ligand complexes, the polar solvation energies ranged between 7.468 and 89.056 kJ/mol (Table 3). M^pro^-Remdesivir demonstrated the highest polar energy of 89.056 kJ/mol, while M^pro^-ZINC000055656943 had the lowest (7.468 kJ/mol). For the RBD–ligand complexes, polar solvation energies ranging from 14.435 to 80.060 kJ/mol were observed (Table 3). RBD-Remdesivir demonstrated the highest polar solvation energy of 80.060 kJ/mol.

#### 2.5.1. Energy Decomposition per Residue

MM/PBSA computations are used to decompose calculated free energies either by per-residue or pair-wise decompositions [90,94]. The per-residue decomposition involves the decomposition of each residue by including the interactions in which one residue atom is involved. Alternatively, pair-wise decomposition interactions can be decomposed by specific residue pairs by including only those interactions in which one atom from each of the analyzed residues is participating [90,94]. These techniques provide useful insight into important interactions of key residues in free energy contribution. Residues contributing binding free energy greater than 5 kJ/mol or less than −5 kJ/mol are worthy of consideration as key residues for the binding of a ligand to a protein [95].

##### Per-Residue Energy Decomposition of M^pro^–Ligand Complexes

**Figure 4:**
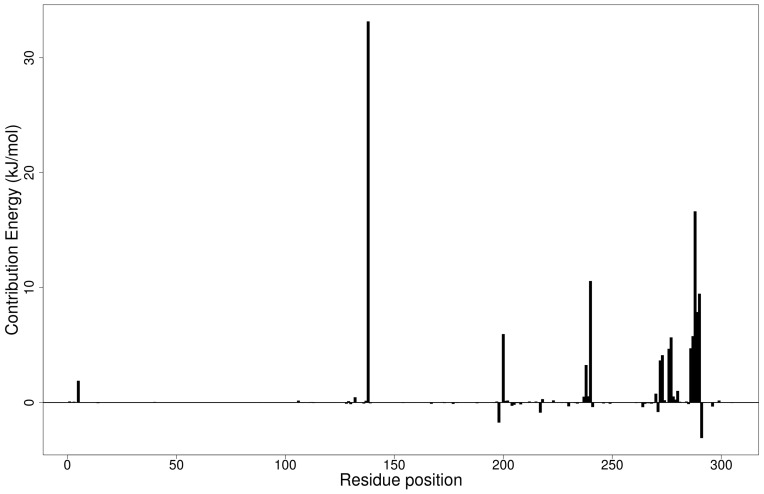
Molecular mechanics/Poisson-Boltzmann surface area (MM/PBSA) plot of binding free energy contribution per residue of the M^pro^-ZINC000095486008 complex.

Generally, amino acid residues within the range of 230 to 290 were observed to contribute energies beyond the ±5 kJ/mol threshold. For the M^pro^–ligand complexes, Tyr237, Tyr239 and Leu272 were common residues that contributed energies greater than 5 kJ/mol or lesser than 5 kJ/mol (Figure 4 and Figure S5A–G). For the M^pro^-NANPDB2245 complex, Tyr237, Tyr239, Leu271, Leu272 and Ala285 were involved in the protein–ligand interaction with individual residue energies of 9.4613, 7.4232, 5.8197, 8.5205, and 5.4298 kJ/mol, respectively (Figure S5B). Lys236, Tyr237, Asn238, Tyr239 and Leu272 contributed energies of 9.4957, 10.5023, 5.0114, 9.6197 and 10.2874 kJ/mol, respectively, in the M^pro^-fusidic acid complex (Figure S5D). For the M^pro^-Remdesivir, only Tyr237 was observed to contribute energy beyond the ±5 kJ/mol threshold (−5.5271 kJ/mol) (Figure S5A).

The M^pro^-ZINC000095486008 complex had the greatest number of residues contributing energies to the interaction (Figure 4). Lys137 contributed the highest energy (33.1602 kJ/mol), followed by Leu287 (16.6352 kJ/mol). Thr199, Tyr239, Met276, Leu286, Glu288 and Asp289 also contributed individual energies of 5.9548, 10.5721, 5.6664, 5.7757, 7.8710 and 9.4673 kJ/mol, respectively (Figure 4). In a previous study, ZINC000095486008 in a complex with Ebola virus viral protein 24 (EBOV VP24) also had high interaction energies per residue [45]. Only Lys137 contributed beyond the threshold (10.7331 kJ/mol) in the M^pro^-ZINC000055656943 complex (Figure S5E). The interaction between ZINC001645993538 and M^pro^ caused residues Tyr237, Tyr239, Leu271, Leu272, Gln273 and Gly275 to contribute energies of 9.7242, 7.5793, 5.6107, 15.8221, 8.4805 and 5.0989 kJ/mol, respectively (Figure S5F). The interaction with oxymetholone also involved Ser46 and Gln189 with energy contributions 8.2579 and 12.2857 kJ/mol, respectively (Figure S5G).

##### Per-Residue Energy Decomposition of the RBD–Ligand Complexes

For the RBD–ligand complexes, residues within the range 340–375 were observed to be involved in interactions contributing energies beyond ±5 kJ/mol (Figure S5H–O). Only Tyr505 showed energy beyond ±5 kJ/mol for the RBD-Remdesivir complex (−5.9154 kJ/mol) (Figure S5H). Residues from RBD complexes of NANPDB2245, ZINC000095486008 and ZINC001645993538 were observed to contribute individual energies beyond the ±5 kJ/mol thresholds (Figure S5I,L,N). For the RBD-NANPDB2245 complex, Asn343 and Ser373 were observed to contribute 5.3285 and 5.286 kJ/mol, respectively (Figure S5I). Leu368 and Phe374 contributed −5.0598 and −5.3661 kJ/mol, respectively, in the RBD-ZINC000095486008 complex (Figure S5L). Additionally, Leu335, Asp364 and Val367 contributed energies of −6.3651, 5.5776 and −7.2196 kJ/mol in the RBD-ZINC001645993538 interactions, respectively, (Figure S5N). These residues need to be investigated to ascertain their critical roles in RBD–ligand binding.

### 2.6. Molecular Dynamics

To further understand the dynamic behavior of the hits within the active sites of the protein structures, 100 ns MD simulations were performed for the unbound protein structures and two selected protein–ligand complexes (NANPDB2403 and ZINC95486008) in duplicates. The root mean square deviation (RMSD), the root mean square fluctuations (RMSF), and the radius of gyration (Rg) were assessed. In addition, graphs for the 10 ns MD simulations were provided in Figure S4.

#### 2.6.1. Root Mean Square Deviation of the Complexes for 100 ns MD Simulations

**Figure 5:**
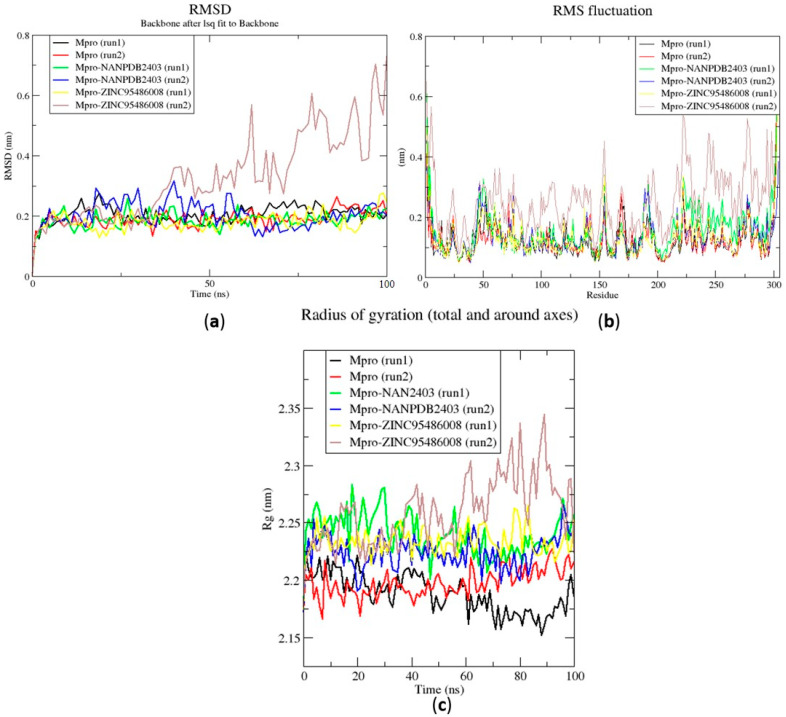
Root mean square deviation (RMSD), root mean square fluctuations (RMSF), and the radius of gyration (Rg) plots of the extended molecular dynamics (MD) simulations of the M^pro^–ligand complexes generated over 100 ns using GROMACS. (**a**) RMSD versus a time graph of the M^pro^–ligand, (**b**) analysis of RMSF trajectories of residues of the M^pro^–ligand, and (**c**) the Rg versus a time graph of the M^pro^–ligand complexes. In all the three graphs, the unbound M^pro^, unbound M^pro^ duplicate run, Mpro-NANPDB2403, Mpro-NANPDB2403 duplicate run, M^pro^-ZINC000095486008, and M^pro^-ZINC000095486008 duplicate run are represented as black, red, green, blue, yellow and brown, respectively.

**Figure 6:**
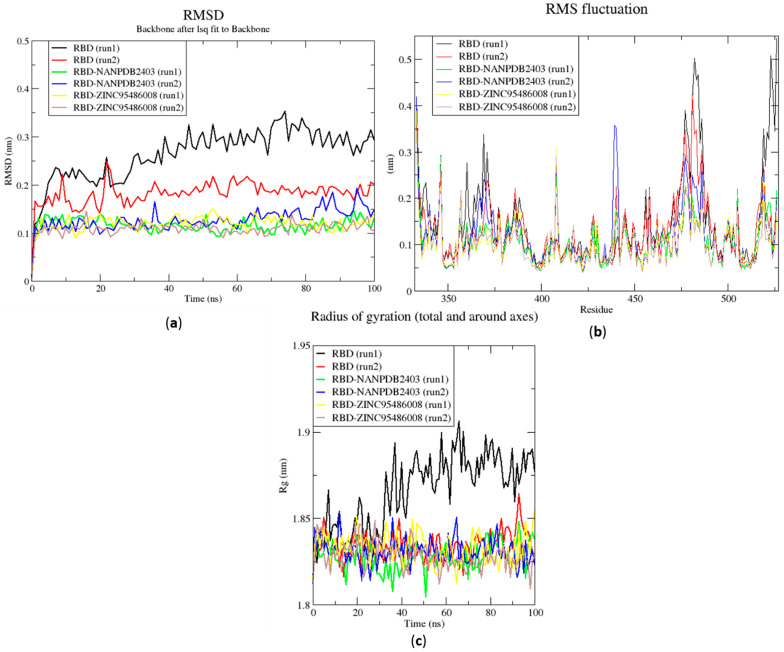
RMSD, RMSF and Rg plots of the extended MD simulations of the RBD–ligand complexes generated over 100 ns using GROMACS. (**a**) RMSD versus time graph of the RBD–ligand complexes, (**b**) analysis of RMSF trajectories of residues of the RBD–ligand complexes, and (**c**) Rg versus time graph of the RBD–ligand complexes. In all the three graphs, the unbound RBD, unbound RBD duplicate run, RBD-NANPDB2403, RBD-NANPDB2403 duplicate run, RBD-ZINC000095486008, and RBD-ZINC000095486008 duplicate run are shown as black, red, green, blue, yellow and brown, respectively.

The RMSD is a plausible measure of protein stability. For the unbound protein, its RMSD sharply rose to 0.2 nm and plateaued around this figure during the simulation (Figure 5a). A similar occurrence was observed for the duplication run. Both runs for the Mpro-NANPDB2403 complex showed similar RMSD, with little deviations (Figure 5a). The duplicate run of the M^pro^-ZINC000095486008 complex was observed to have a stable RMSD with an average value of 0.2 nm until about 30 ns, where a rise was observed until the end of the 100 ns period (Figure 5a). In all the structures, the RMSDs were observed to fluctuate within the range of 0.13–0.32 nm (except the duplicate run of M^pro^-ZINC000095486008) (Figure 5a). A recent study virtually screened FDA-approved antiviral drugs against the M^pro^ and performed 100 ns molecular dynamics simulations of protein–ligand complexes [96]. The study reported an RMSD range of 1.5 Å (0.15 nm) to 3 Å (0.3 nm), with an average RMSD of 2.25 Å (0.225 nm) for all complexes [96], consistent with the RMSD range reported (Figure 6a) and other studies [97,98].

The RMSD values of the RBD and RBD–ligand complexes were also computed. For the unbound RBD, the RMSD averaged between 0.2 nm and 0.25 nm during the first 20 ns of the simulation (Figure 6a). It then rose to about 0.3 nm, where it was fairly stable during the rest of the simulation. The RMSD of the unbound protein averaged around 0.15 nm (Figure 6a). For both runs, the RBD-ZINC95486008 complex showed the most stable RMSD, with both averaging around 0.1 nm. During the two duplicate runs, the RBD-ZINC000095486008 complex maintained an average RMSD of 0.125 nm throughout the 100 ns simulation period (Figure 6a). Generally, the unbound RBD structures had the highest RMSD values (Figure 6a). A recent molecular dynamics study of RBD and terpene compounds reported a similar RMSD range of 0.08 and 0.25 nm [99].

#### 2.6.2. Root Mean Square Fluctuation of the Complexes for 100 ns MD Simulations

The RMSF was computed to analyze the residual fluctuations over the simulation time. For the M^pro^ and its complexes, fluctuations were observed at regions from residue indexes of 50–55, 150–160, 215–230 and 275–280 (Figure 5b). For the RBD, fluctuations were observed in all the RBD–ligand complexes around regions 420–430, 470–480 and 520–530 (Figure 6b). A few residues in the range 470–480 showed more flexibility in the RBD for both duplicate runs than the RBD-NANPDB2403 and RBD-ZINC95486008 complexes (Figure 6b). The results obtained corroborate with that of a previous study that identified SARS-CoV-2 RBD inhibitors via molecular docking and dynamics simulations studies of terpenes [99].

#### 2.6.3. Radius of Gyration of the Complexes for 100 ns MD Simulations

The radius of gyration was assessed to evaluate the compactness of the structures. A stably folded protein maintains a reasonably steady Rg over the simulation time. Considering M^pro^, the Rg of the unbound M^pro^ for both duplicate runs was relatively steady and averaged around 2.2 nm (Figure 5c). The duplicate run of the M^pro^-ZINC000095486008 was observed to have a similar Rg as the other, maintaining an average Rg value of 2.25 nm until about 30 ns, where it spiked and then fluctuated throughout the rest of the simulation time (Figure 5c). For the duplicate runs, M^pro^-NANPDB2403 had a steady Rg over the simulation and averaged around 2.25 nm. Previous MD simulation studies of M^pro^ in complex with lichen spp. compounds revealed Rg values ranging between 2.175 nm and 2.25 nm [98], consistent with the Rg values reported elsewhere [100] and in this work. 

The Rg curves of the unbound RBD and RBD–ligand complexes for 100 ns ranged between 1.8 and 1.91 nm (Figure 5c), consistent with the Rg ranges for the 10 ns MD simulations and in a recent study (1.8 and 1.88 nm) [99]. The unbound RBD had similar Rg with that of the duplicate, with an average of 1.84 nm until 30 ns, where it increased gradually to an average of 1.88 nm until the end of the 100 ns period (Figure 5c).

### 2.7. Comparison of Binding Modes Pre-MD and Post-100 ns MD Simulations 

**Figure 7:**
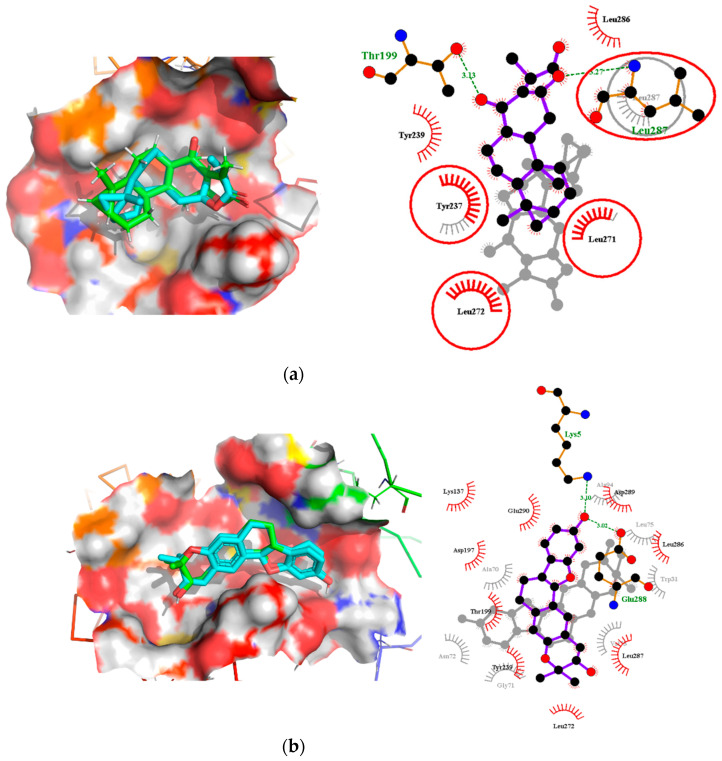
Binding mode characterization using superimposition and LigPlot+ analyses. (**a**) The pre- and post-100 ns MD simulations of M^pro^-NANPDB2403, and (**b**) M^pro^-ZINC95486008 complexes. Ligands for the pre- and post-MD simulations are shown in green and cyan colors, respectively. The overlapped interactions between the pre- and post-MD simulations are circled in red color.

**Figure 8:**
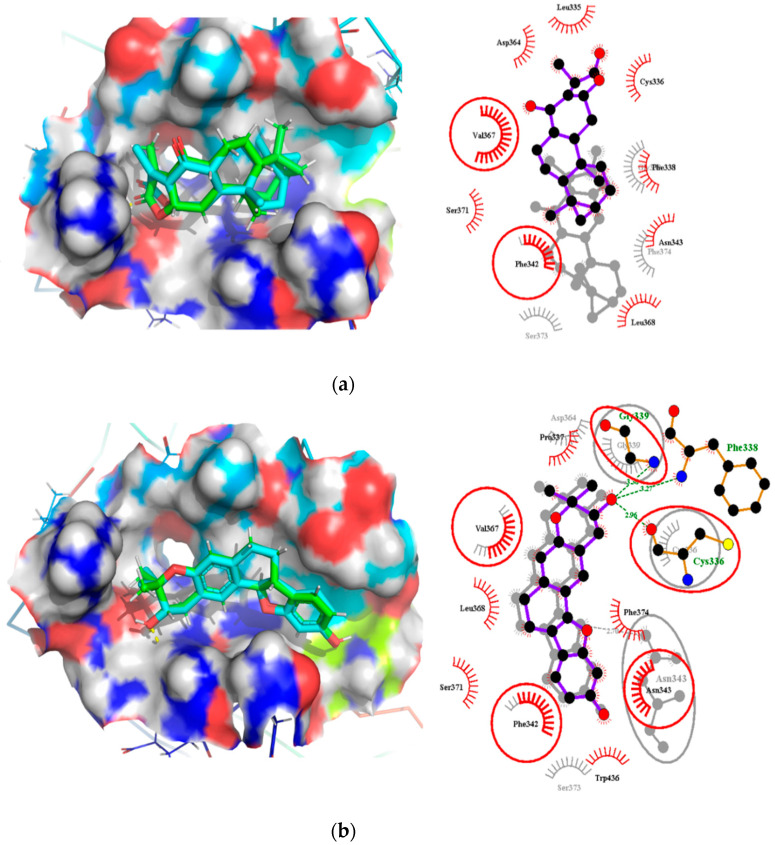
Binding mode characterization using superimposition and LigPlot+ analyses. (**a**) The pre- and post-100 ns MD simulations of RBD-NANPDB2403, and (**b**) RBD-ZINC95486008 complexes. Ligands for the pre- and post-MD simulations are shown in green and cyan colors, respectively. The overlapped interactions between the pre- and post-MD simulations are circled in red color.

Analyses of the binding modes of potential leads NANPDB2403 and ZINC95486008 in complex with both target structures (M^pro^ and RBD) were undertaken after both docking and 100 ns MD simulation. This was to ascertain if the same binding modes were maintained after undergoing MD simulation. Binding mode superimpositions and visual inspections revealed that the compounds resided well in the active site pocket of each target, with almost the same binding modes for the pre-MD docked complex and post-MD simulations results (Figure 7 and Figure 8). The evaluation of the binding modes based on the superimposition of the complexes gave RMSD values for NANPDB2403 and ZINC95486008 in M^pro^ pre-MD and post-MD as 1.167Å and 0.703Å, respectively. For the RBD target, RMSD values 0.807Å and 1.396Å were obtained for both compounds, which were less than 2Å, considered as the threshold for good alignment [101]. Therefore, the binding poses could be considered similar even after MD simulations.

#### 2.7.1. Binding Modes Interactions Analysis between M^pro^ and Potential Leads

The pre-MD interaction analysis revealed that the NANPDB2403 complex formed hydrogen bond interactions with the Thr199 and Leu287 residues. However, after MD simulations, these hydrogen bond interactions were lost. Instead, the ligand formed hydrophobic interactions with Leu287 and no interactions with Thr199. Likewise, the pre-existing hydrophobic bonds were only maintained for Tyr237, Leu271 and Leu272. The hydrophobic bond interactions for Tyr237 and Tyr239 were lost after the simulation (Table 4, Figure 7a and Figure S2A,B). 

ZINC95486008 formed only hydrophobic interactions before and after MD simulations. Surprisingly, the hydrophobic interactions observed after MD simulations were completely different from those observed before. New hydrophobic interactions were found between the ligand and residues Trp31, Ala70, Gly71, Asn72, Val73, Leu75 and Ala94 (Table 4, Figure 7b and Figure S2C,D). Both compounds docked well within the active site after MD simulations.

#### 2.7.2. Binding Modes Interactions Analysis between RBD and Potential Leads

For the RBD target, hydrogen bond interactions were not observed for NANPDB2403 ligand before and after MD simulations. However, two residues namely Phe342 and Val367 maintained their hydrophobic interactions after the simulation. New interactions were observed between the ligand and residues Gly339, Ser373 and Phe374 (Table 5, Figure 8a and Figure S2E,F).

The interactions for ZINC95486008 and the target before the simulation showed that hydrogen bond interactions formed between the ligand and residues Cys336, Phe338 and Gly339. Even though, Cys336 has been reported as a critical residue for binding, it did not maintain the hydrogen bond after MD simulations but rather formed a hydrophobic contact. It is worth noting that after MD simulations, ZINC95486008 formed a hydrogen bond with Asn343, a residue predicted as a critical. Residues whose hydrophobic bonds were maintained after MD simulations include Phe342 and Val367. However, ZINC95486008 formed hydrophobic bonds with Gly339, a residue that previously formed a hydrogen bond during pre-MD simulation (Table 5, Figure 8b and Figure S2G,H). 

### 2.8. Summary and Implications of the Results

Six potential anti-SARS-CoV-2 biomolecules were identified as leads comprising NANPDB2245, NANPDB2403, fusidic acid, ZINC000095486008, ZINC0000556656943 and ZINC001645993538 (Table 6). These molecules were obtained by screening libraries made up of ANC, known drugs and machine learning-derived compounds against the SARS-CoV-2 receptors M^pro^ and RBD. The known drugs included antiviral remdesivir, dexamethasone, hydroxychloroquine and chloroquine. The techniques utilized included previously described methods used to identify potential bioactive compounds against the Ebola virus protein VP24 [45]. 

Fusidic acid was shown to be structurally similar to NANPDB2245 and NANPDB2403 via DrugBank. Betulinic acid was also identified as structurally similar to oxymetholone. In our previous study, helioscopinolide C which is a structural analog of NANPDB2245 (helioscopinolide B), and ZINC000095486008 were reported as being plausible anti-Ebola compounds [45]. These compounds warrant further in vitro and in vivo testing to ascertain their anti-SARS-CoV-2 activity. The results also corroborate ongoing research on the inhibitory activities of remdesivir and oxymetholone. Since fusidic acid is already an FDA-approved drug for humans, this offers the opportunity to explore its therapeutic potential against SARS-CoV-2. 

The study proposed potential anti-SARS-CoV-2 compounds, which were reinforced with antiviral activity predictions. Additionally, the study highlights the repurposing of existing drugs as potential anti-SARS-CoV-2 molecules. This study complements ongoing efforts geared towards the identification of SARS-CoV-2 inhibitors. Making these predicted compounds accessible to the scientific community could stimulate the pace of searching for effective SARS-CoV-2 drugs.

## 3. Materials and Methods

**Figure 9:**
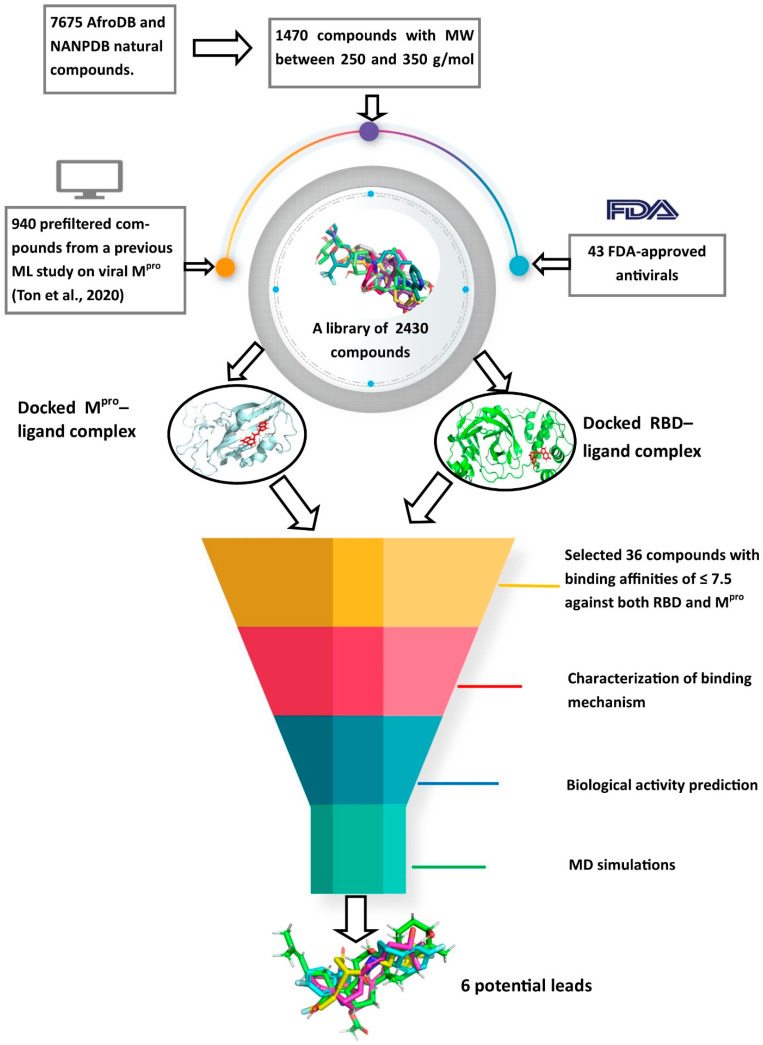
Methodology schema employed in the cheminformatics studies for predicting potential anti-SARS-COV-2 compounds. Natural compounds from the African Natural Product Database (AfroDB), North African Natural Product Database (NANPDB), the Food and Drug Administration (FDA)-approved antivirals and compounds from previous machine learning (ML) studies were docked against SARS-CoV-2 receptors M^pro^ and RBD. Molecular weights (MW) were used to prefilter the molecules. Compounds with binding affinities of ≤−7.5 kcal/mol against both receptors were selected for the downstream analysis. Methods include the characterizations of the protein–ligand complexes, biological activity predictions and MD simulations.

A schema showing the step-by-step techniques employed in predicting the potential leads is shown in Figure 9. A library consisting of ANC, FDA-approved drugs and machine learning-derived compounds were screened against the structures of SARS-CoV-2 receptors M^pro^ and RBD of the spike protein. The compounds were prefiltered using molecular weights (MW) between 250 and 350 g/mol, good absorption, distribution, metabolism, excretion and toxicity (ADMET) profiles. The docked complexes were subjected to MD simulations, and the biological activities of the hits were predicted.

### 3.1. Data Sources for SARS-CoV-2 Targets

The experimentally solved 3D structures of SARS-CoV-2 M^pro^ and RBD of the spike protein (Accession numbers: PDB IDs 5R82 and 6M0J, respectively) were retrieved from the Research Collaboratory for Structural Bioinformatics Protein Data Bank (RCSB PDB) [102]. 5R82 is a monomer of the SARS-CoV-2 M^pro^ with a co-crystallized ligand (6-(ethylamino) pyridine-3-carbonitrile), while 6M0J is also a monomer of the SARS-CoV-2 RBD in complex with the human ACE2.

### 3.2. The Screening Library

A library of 7675 compounds was created from the North African Natural Product and African Natural Product Databases [42,43]. The natural compounds were filtered using ADMET Predictor™ (V8.0, Simulations Plus, Inc., Lancaster, PA, USA) and those with high toxicity levels and molecular weights greater than 350 g/mol and less than 250 g/mol were eliminated [45,103], which reduced the number to 1470. Additionally, the 1000 top hit compounds generated by a machine learning (ML) study that screened 1.3 billion compounds against the M^pro^ [28] were also screened based on Lipinski’s rule of five using OpenBabel [104]. A total of 60 compounds were eliminated and the remaining 940 added to the library. Additionally, a set of 43 FDA-approved antivirals, including those undergoing clinical trials [105], were added to the library. Thus, the library used for molecular docking was composed of 2453 compounds.

### 3.3. Preparation of the Protein Structure and Elucidation-Binding Sites

The 3D protein structures of M^pro^ and RBD were analyzed using PyMOL Version 1.5.0.4 (PyMOL Molecular Graphics System, Schrödinger, LLC) as described [106]. The structures were first cleaned of all available water molecules and ligands before being subjected to energy minimization. A 10 ns molecular dynamics (MD) simulation for each of the structures was performed using the Groningen Machine for Chemical Simulations GROMACS version 2018 [107]. The Optimized Potentials for Liquid Simulations (OPLS)/All Atom (AA) force field was used to generate the protein topologies and position restrain files. Periodic boundary conditions (PBC) were applied to each structure, with the protein centered 1 nm from the edge of a cubic box to monitor the movement of all particles and avoid edge effects on the surface atoms [108]. The system was solvated with SPC water and neutralized, and the steepest descent algorithm used for the energy minimization was at 50,000 steps. A 100 ps equilibration simulation was performed using the NVT and NPT ensembles to ensure that the system was well-equilibrated to an optimal temperature of 300 K and pressure of 1 bar before the MD simulation, which was performed for 10 ns. Xmgrace was used to generate the graphical outputs [109]. The binding sites of the proteins were then predicted using the Computed Atlas of Surface Topography of proteins (CASTp) version 3.0 (available at http://sts.bioe.uic.edu/castp/calculation.html), which utilizes theoretical and algorithmic results of computational geometry to predict pockets and cavities, whiles excluding shallow depressions [49]. The predicted binding cavities were visualized and analyzed using Chimera version 1.12 and PyMOL. Predicted sites with very small volumes and areas such that no ligands could fit were excluded from downstream virtual screening.

### 3.4. Virtual Screening of Ligand Library

AutoDock Vina [61] was used to screen the integrated library against the energy-minimized M^pro^ and RBD. The library was imported into the OpenBabel workspace [104] in “.sdf” file format and minimized using the Universal Force Field (Uff) for 200 steps and then optimized using the conjugate gradient before finally converting the file into the AutoDock format (“.pdbqt”). The file format “.pdbqt” stores the atomic coordinates, partial charges, and describes the rigid and rotational parts of the molecule, as well as serves as the input format for AutoDock Vina. AutoDock Vina employs an empirical and knowledge-based scoring function to predict the binding affinity of compounds [61]. The specified dimensions of the grid box for the M^pro^ were 37.58 * 64.78 * 62.77 Å^3^ and centered at 49.33, 49.36, and 49.56 Å. The corresponding dimensions for the RBD were 42.54 * 42.73 * 42.44 Å^3^ and centered at 41.19, 47.74, and 55.37 Å. The grid boxes covered all the predicted binding pockets of the two proteins. The compounds with binding energies of −7.5 kcal/mol or less for both receptors were selected for downstream analysis [62].

### 3.5. Characterization of the Protein–Ligand Interactions

LigPlot+ [69] was used to generate the 2D protein–ligand interactions which revealed hydrogen and hydrophobic interactions. The best poses of the hits were saved in “.pdb” file format and then visualized in PyMol [106]. The saved protein–ligand complexes served as inputs for LigPlot+ [69]. The hydrogen bonds were denoted as green dashed lines and the arcs with spokes radiating towards the ligands as the hydrophobic interactions. Default parameters were used in generating the interaction profiles.

### 3.6. Prediction of Antiviral Properties of Hit Compounds

The antiviral activities of the hits were predicted using the Bayesian-based Prediction of Activity Spectra for Substances (PASS) [110] using the Simplified Molecular Input Line-Entry System (SMILES) format of the compounds as inputs. PASS determines the relevant biological activities of compounds based on the structural–activity relationship between the compound of interest and a training set of over 26,000 compounds with known biological activities [110]. For any given compound, PASS predicts the Pa and Pi, with both ranging between 0.000 and 1.000 for a predicted activity. When the Pa is greater than the Pi for a particular compound activity and Pa > 0.3, it is worth exploring the pharmacological activity [111,112]. PASS was used in previous studies to predict the antiviral activity of novel compounds, and the experimental results corroborated the PASS predictions [113].

### 3.7. Molecular Dynamics Simulation of Protein–Ligand Complexes

MD simulations of the protein–ligand complexes were performed using GROMACS 2018 [107]. Their protein topologies were generated using the CHARMM36 all-atom force field. The ligand topologies were generated using the CHARMM force field via the CHARMM General Force Field (CGenFF) server (available at https://cgenff.umaryland.edu/). Complexes were generated from the ligands and protein topologies for each of the selected cases under study. Each complex was solvated with the transferable intermolecular potential with a 3 points (TIP3P) water model in a cubic box of size 1.0 nm and neutralized with Na and Cl ions. Energy minimization of each complex was conducted for 50,000 steps using the steepest descent algorithm. The ligands were restrained before the NVT and further using the NPT ensemble. Equilibration of each complex was performed for 100 ps apiece and the final MD simulation was conducted for 10 ns with time steps of 2 fs under the PME. Extended MD runs (in duplicates) were also conducted for 100 ns for the unbound proteins and selected protein–ligand complexes of NANPDB2403 and ZINC95486008. Duplicate MD runs were carried out by generating random seeds for the initial velocities of each run.

The root mean square deviation (RMSD), root mean square fluctuation (RMSF), and radius of gyration (Rg) of the unbound proteins and selected protein–ligand complexes were determined. RMSD is a frequently used measure of the differences between the structures sampled during the simulation and the reference structure [114]. MD simulations require systems to be close to their equilibrium (native) conformation. The time trajectory of RMSD shows how a protein structure deviates from a reference structure as a function of time [114].

RMSF measures the movement of a subset of atoms concerning the average structure over the entire simulation. RMSF indicates the flexibility of different regions of a protein, which can be related to crystallographic B factors [114]. Residues contributing to the complex structural fluctuation can be assessed using this stability profile analysis. Higher RMSF values imply greater fluctuations. Greater amounts of structural fluctuations occur in regions known to be involved in ligand binding and catalysis, notably the catalytic loop regions [115]. Adaptive variation in flexibility lies principally in these regions of the sequence that influence the conformational stabilities of the protein–ligand complex [115].

The radius of gyration (Rg) assesses the changes in compactness of a protein–ligand complex over the simulation time. The loss of compactness affects the stability of the complex by introducing weak intermolecular bonds. When the Rg of a complex is relatively steady, the compactness of the protein–ligand complex is high, and the protein is folded well, whereas the Rg value changes over time if the protein unfolds [45,116].

G_mmpbsa [48] was also used to calculate the free binding energies of each complex over the 10 ns simulation period, utilizing frames in steps of 0.1 ns. The binding free energy contribution per residue was calculated using the MM/PBSA and the plots generated using R programming.

### 3.8. Analysis of Binding Modes

From the aforementioned, two out of six lead compounds comprising NANPDB2403 and ZINC95486008 were selected for an extended 100 ns MD simulation for both targets, and their binding modes were analyzed comparatively for the pre- and post-MD simulations. Binding mode analysis was done based on whether the compounds maintained their poses and interactions with the residues in the binding site pockets after the simulations. This was evaluated using RMSD resulting from superimposition of the complexes, visual inspection and intermolecular interactions via LigPlot+.

## 4. Conclusions

The study utilized cheminformatics techniques to identify six potential anti-SARS-COV-2 compounds from an integrated compound library made up of natural products from Africa, machine learning-based studies and drugs undergoing clinical trials. The compounds were screened against the binding pockets of two putative drug targets, namely RBD of the spike protein and the M^pro^. The six potential lead compounds, namely NANPDB2245, NANPDB2403, fusidic acid, ZINC000095486008, ZINC0000556656943 and ZINC001645993538 had binding energies ranging from −6.9 kcal/mol to −8.2 kcal/mol and 7.2 kcal/mol to −8.0 kcal/mol against the M^pro^ and RBD of spike protein, respectively. Additional molecular dynamics simulations coupled with MM/PBSA calculations reinforced the potential inhibition of the two SARS-CoV-2 therapeutic targets. These identified druglike biomolecules have good pharmacological profiles with insignificant toxicity. The compounds were also predicted to have a high propensity in inhibiting viral entry and replication. The predicted scaffolds could form the basis for the de novo design of the next-generation SARS-CoV-2 compounds for clinical evaluations. Potentially novel critical binding residues were identified that could help in the design of new inhibitors. The studies are computational and would therefore need in vitro studies to corroborate the findings.

## Figures and Tables

**Table 1:** Predicted binding sites in the viral main protease (M^pro^) and receptor-binding domain (RBD) via CASTp, including the dimensions of the volumes and areas.

Pocket	Pocket Area (Å^2^)	Volume (Å^3^)	Residues Lining Pockets
**M^pro^**			
1	557.1	920.6	Thr24, Thr25, Thr26, Leu27, His41, Cys44, Thr45, Ser46, Met49, Leu50, Phe140, Leu141, Asn142, Gly143, Ser144, Cys145, His163, His164, Met165, Glu166, Leu167, Pro168, Asp187, Arg188, Gln189, Thr190, Gln192
2	380.0	465.1	Met6, Ala7, Phe8, Pro9, Gly11, Lys12, Val13, Gln127, Phe150, Ile152, Asp153, Tyr154, Val157, Phe291, Asp295, Arg298, Gln299, Val303, Thr304
3	107.5	151.3	Phe3, Arg4, Lys5, Trp207, Leu282, Ser284, Glu288, Phe291
4	193.5	225.2	Pro108, Gly109, Gln110, Pro132, Ile200, Thr201, Val202, Asn203, Glu240, His246, Ile249, Thr292, Pro293, Phe294
5	86.6	131.4	Glu14, Gly15, Met17, Val18, Trp31, Ala70, Gly71, Val73, Asn95, Lys97
**RBD**			
A	148.3	169.2	Arg454, Phe456, Arg457, Lys458, Asp467, Ser469, Glu471, Ile472, Tyr473, Pro491
B	52.3	90.3	Phe342, Asn343, Leu368, Ser371, Ser373, Phe374
C	146.8	160.6	Glu340, Val341, Ala344, Arg346, Phe347, Ala348, Asn354, Arg355, Lys356, Ala397, Ser399, Val511

**Table 2:** The binding energies and intermolecular interactions between selected compounds and M^pro^ as well as RBD. Compounds are from the African Natural Compounds (ANC) database and Machine Learning Study (ML). In addition, antivirals and experimental drugs are included.

Compound	Source	Binding Energy (kcal/mol)		Hydrogen Bonds [Bond Length (Ȧ)]		Hydrophobic Bonds	
		M^pro^	RBD	M^pro^	RBD	M^pro^	RBD
**Selected Hits**							
NANPDB2403	ANC	−8.1	−7.8	Leu287 (3.22)	-	Thr199, Tyr237, Tyr239, Leu271, Leu272, Leu286.	Leu335, Cys336, Phe338, Phe342, Asn343, Asp364, Val367, Leu368, Ser371,
NANPDB2245	ANC	−8.0	−7.7	Arg131 (2.92)	Asn343 (2.96)	Lys137, Thr199, Tyr237, Tyr239, Leu271, Leu272, Leu286, Leu287, Asp289	Leu335, Cyc336, Phe338, Gly339, Asp364, Val367, Leu368, Ser371, Phe374
ZINC000055656943	ML	−8.0	−8.0	Asp197 (2.80)	-	Arg131, Thr198, Thr199, Tyr237, Tyr239, Leu272, Leu287	Leu335, Cys336, Phe338, Phe342, Asp364, Val367, Leu368, Ser371, Phe374,
ZINC000095486008	ANC	−8.2	−7.8	Lys5 (3.1), Glu288 (3.02)	Cys336 (2.96), Phe338 (3.26), Gly339 (3.3)	Lys137, Asp197, Thr199, Tyr239, Leu272, Leu286, Leu287, Asp289, Glu290	Pro337, Phe342, Asn343, Val367, Leu368, Ser371, Phe374, Trp436
ZINC001645993538	ML	−7.7	−7.5	Thr199 (314)	-	Lys137, Asp197, Tyr239, Leu272, Leu286, Leu287, Glu288, Asp289	Cys336, Phe338, Asp364, Val367, Leu368, Ser371, Phe374
**Known Antivirals and Experimental Drugs**							
Oxymetholone		−7.8	−7.7	Thr25 (2.81), Glu166 (2.9, 3.00)	Cys336 (3.0), Asn343 (3.09)	His41, Ser46, Thr45, Asn142, Gly143, Cys145, His164, Met165	Leu335, Phe338, Gly339, Phe342, Asp364, Val367, Leu368, Ser371, Phe374
Dexamethasone		−7.6	−6.7	Asp197 (2.9, 3.24), Met276 (3.01), Leu287 (3.29, 3.32)	Arg355 (2.99, 3.01), Thr430 (3.18), Glu516 (2.76)	Lys137, Thr198, Thr199, Tyr239, Leu271, Gly275, Leu286, Leu287, Asp289	Pro426, Phe429, Pro463, Phe464, Phe515
Remdesivir		−6.8	−6.3	Lys137 (3.19), Thr199 (2.82, 3.05), Leu287 (3.09), Asp289 (2.84)	Gly496 (2.84, 2.96), Asn501 (2.9)	Arg131, Asp197, Thr198, Tyr237, Asn238, Tyr239, Leu271, Leu272, Asn274, Gly275, Met276, Leu286	Arg403, Tyr453, Leu455, Ser494, Tyr495, Phe497, Tyr505
Hydroxychloroquine		−5.9	−5.5	Asp197 (3.05, 3.22), Thr199 (3.26)	Thr345 (2.97), Asn354 (3.04), Ala397 (2.72), Ser399 (2.99),	Arg131, Thr198, Tyr237, Tyr239, Leu272, Met276, Ala285, Leu286, Leu287, Asp289	Glu340, Val341, Ala344, Arg346, Phe347, Ala348, Arg355, Lys356, Asp398
Chloroquine		−5.5	−4.9	Tyr239 (3.2)	-	Arg131, Asp197, Thr198, Thr199, Tyr237, Leu272, Leu286, Leu287, Asp289,	Arg403, Tyr449, Tyr453, Ser494, Tyr495, Gly496, Phe497, Asn501, Tyr505

**Table 3:** Contributing energy terms of the Molecular Mechanics/Poisson-Boltzmann Surface Area (MM/PBSA) computations for receptor–ligand complexes. Values are shown as average ± standard deviations in kJ/mol. The terms consist of van der Waals, electrostatic, polar solvation, solvent-accessible surface area (SASA) and binding energies.

Compound	van der Waals Energy (kJ/mol)	Electrostatic Energy (kJ/mol)	Polar Solvation Energy (kJ/mol)	SASA Energy (kJ/mol)	Binding Energy (kJ/mol)
**M^pro^**					
NANPDB2245	−85.61 +/− 11.970	−6.274 +/− 7.537	46.495 +/− 10.814	−10.829 +/− 1.110	−56.223 +/− 11.988
NANPDB2403	−77.965 +/− 12.063	−6.624 +/− 7.992	36.397 +/− 13.775	−9.939 +/− 1.139	−58.132 +/− 13.000
ZINC000095486008	−98.620 +/− 15.067	−20.464 +/− 14.240	84.718 +/− 29.042	−12.692 +/− 1.538	−47.058 +/− 20.877
ZINC000055656943	−18.966 +/− 26.649	−2.907 +/− 8.983	7.468 +/− 57.684	−2.692 +/− 4.061	−17.097 +/− 45.262
ZINC001645993538	−84.952 +/− 12.296	−20.470 +/− 13.867	62.338 +/− 24.852	−10.702 +/− 1.140	−53.785 +/− 18.652
Oxymetholone	−60.820 +/− 13.039	−3.207 +/− 5.288	27.787 +/− 20.226	−8.485 +/− 1.967	−44.724 +/− 17.562
Remdesivir	−114.276 +/− 18.798	−19.410 +/− 12.604	89.056 +/− 41.414	−13.726 +/− 2.248	−58.356 +/− 31.051
**RBD**					
NANPDB2245	−30.310 +/− 43.669	−2.337 +/− 4.496	14.435 +/− 40.707	−3.930 +/− 5.769	−22.142 +/− 39.775
NANPDB2403	−79.080 +/− 14.764	−2.714 +/− 7.624	39.552 +/− 18.265	−10.898 +/− 1.698	−53.140 +/− 20.905
ZINC000095486008	−119.217 +/− 10.410	−8.227 +/− 7.728	77.567 +/− 12.472	−15.298 +/− 1.031	−65.174 +/− 10.495
ZINC000055656943	−58.972 +/− 54.205	−11.991 +/− 12.656	34.870 +/− 57.870	−7.003 +/− 6.417	−43.096 +/− 39.685
ZINC001645993538	−109.967 +/− 10.090	−0.990 +/− 6.308	63.10 +/− 8.655	−13.921 +/− 0.760	−61.778 +/− 9.594
Oxymetholone	−109.874 +/− 9.028	−15.240 +/− 7.816	74.123 +/− 15.363	−13.752 +/− 0.876	−64.742 +/− 14.235
Remdesivir	−100.708 +/− 18.622	−11.616 +/− 11.476	80.060 +/− 24.762	−12.206 +/− 1.981	−44.471 +/− 19.222

**Table 4:** Molecular interactions between potential lead compounds and the M^pro^ target before and after molecular dynamics (MD) simulations.

M^pro^				
	Pre-MD Interactions		Post-MD Interactions (100 ns)	
Compound Name	H-Bond Residues	Hydrophobic Bond Residues	H-Bond Residues	Hydrophobic Bond Residues
NANPDB2403	Thr199, Leu287	Tyr237, Tyr239, Leu271, Leu272, Leu286	-	Tyr237, Leu271, Leu272, Leu287
ZINC95486008	Gly5, Glu288	Lys137, Asp197, Thr199, Tyr239, Leu272, Leu286, Leu287, Asp289, Glu290	-	Trp31, Ala70, Gly71, Asn72, Val73, Leu75, Ala94

**Table 5:** Molecular interactions between potential lead compounds and RBD targets before and after MD simulations.

RBD				
	Pre-MD Interactions		Post-MD Interactions (100 ns)	
Compound Name	H-Bond Residues	Hydrophobic Bond Residues	H-Bond Residues	Hydrophobic Bond Residues
NANPDB2403	-	Leu335, Cys336, Phe338, Phe342, Asn343, Asp364, Val367, Leu368, Ser371	-	Gly339, Phe342, Val367, Ser373, Phe374
ZINC95486008	Cys336, Phe338,Gly339	Pro337, Phe342, Asn343, Val367, Leu368, Ser371, Phe374, Trp436	Asn343	Cys336, Gly339, Phe342, Asp364, Val367, Ser373

**Table 6:** A list of selected compounds with their 2D structures and common/IUPAC names generated using the Marvin suite (http://www.chemaxon.com/).

Ligand ID	Common/IUPAC Name	2D Structure
NANPDB2245	Helioscopinolide B	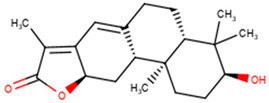
NANPDB2403	Retusolide B	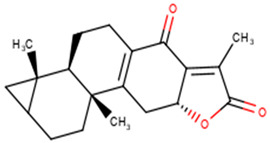
Fusidic acid	Fusidic acid	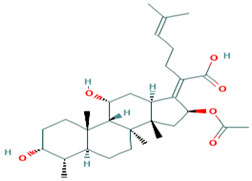
ZINC000095486008	(2R,10R,18S)-17,17-dimethyl-3,16-dioxapentacyclo (1 1.8.0.02,10.04,9.015,20) henicosa-1(13),4,6,8,14,20-hexaene-6,18-diol	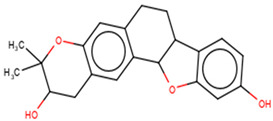
ZINC000055656943	(4S)-7-fluoro-N-((1S)-5-fluoro-2,3-dihydro-1H-inden-1-yl)-2-oxo-1,2,3,4-tetrahydroquinoline-4-carboxamide	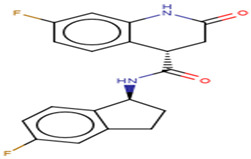
ZINC001645993538	(4S)-N-((1R,3R,6R)-7,7-difluorobicyclo (4.1.0) heptan-3-yl)-7-fluoro-2-oxo-1,2,3,4-tetrahydroquinoline-4-carboxamide	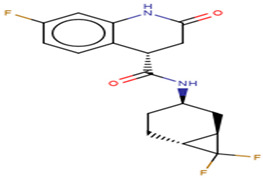

## Data Availability

All the IDs of the datasets used for the work were cited in the manuscript.

## Supplementary Materials

The following are available online. Figure S1: Cartoon representation of RBD in complex with: (a) NANPDB2245 (helioscopinolide B), (b) ZINC000095486008, (c) ZINC001645993538, and (d) Oxymetholone. Figure S2: Binding mode representation and LigPlot+ characterization of: (a) M^pro^ and NANPDB2403 before MD simulation (pre-MD), (b) M^pro^ and NANPDB2403 after MD simulation (post-MD), (c) M^pro^ and ZINC95486008 before MD simulation (pre-MD), (d) M^pro^ and ZINC95486008 after MD simulation (post-MD), (e) RBD and NANPDB2403 before MD simulation (pre-MD), (f) RBD and NANPDB2403 after MD simulation (post-MD), (g) RBD and ZINC95486008 before MD simulation (pre-MD), and (h) RBD and ZINC95486008 after MD simulation (post-MD). Figure S3: Two-dimensional diagram of the RBD–ligand interaction generated using LigPlot+. (a) Interaction profile of the RBD-NANPDB2245 complex, and (b) Interaction profile of the RBD-ZINC000095486008 complex. Figure S4: Graphs of the RMSD, RMSF and radius of gyration of the RBD–ligand complexes generated over a 10 ns molecular dynamics simulation using GROMACS. (a) RMSD versus time graph of the RBD–ligand complexes, (b) Analysis of the RMSF trajectories of the residues of the RBD–ligand complexes, and (c) Rg versus time graph of the RBD–ligand complexes. Figure S5: MM/PBSA plot of the binding free energy contribution per residue of the protein–ligand complexes (a) M^pro^-Remdesivir, (b) M^pro^-NANPDB2245, (c) M^pro^-NANPDB2403, (d) M^pro^-fusidic acid, (e) M^pro^-ZINC000055656943, (f) M^pro^-ZINC001645993538, (g) M^pro^-oxymetholone, (h) RBD-Remdesivir, (i) RBD-NANPDB2245, (j) RBD-NANPDB2403, (k) RBD-fusidic acid, (l) RBD-ZINC000095486008, (m) RBD-ZINC000055656943, (n) RBD-ZINC001645993538, and (o) RBD-Oxymetholone. Table S1: The binding energies and intermolecular interactions between the hits and M^pro^ as well as RBD.

## Sample Availability

Samples of the compounds are not available from the authors.
