# Supplementary material for: Cheminformatics-Based Identification of Potential Novel Anti-SARS-CoV-2 Natural Compounds of African Origin
Source: Molecules. 2021 Jan 14;26(2):406. doi: 10.3390/molecules26020406 (PMC7829843; doi:10.3390/molecules26020406)
Supplement: Supplementary file 1 [file molecules-26-00406-s001.pdf]

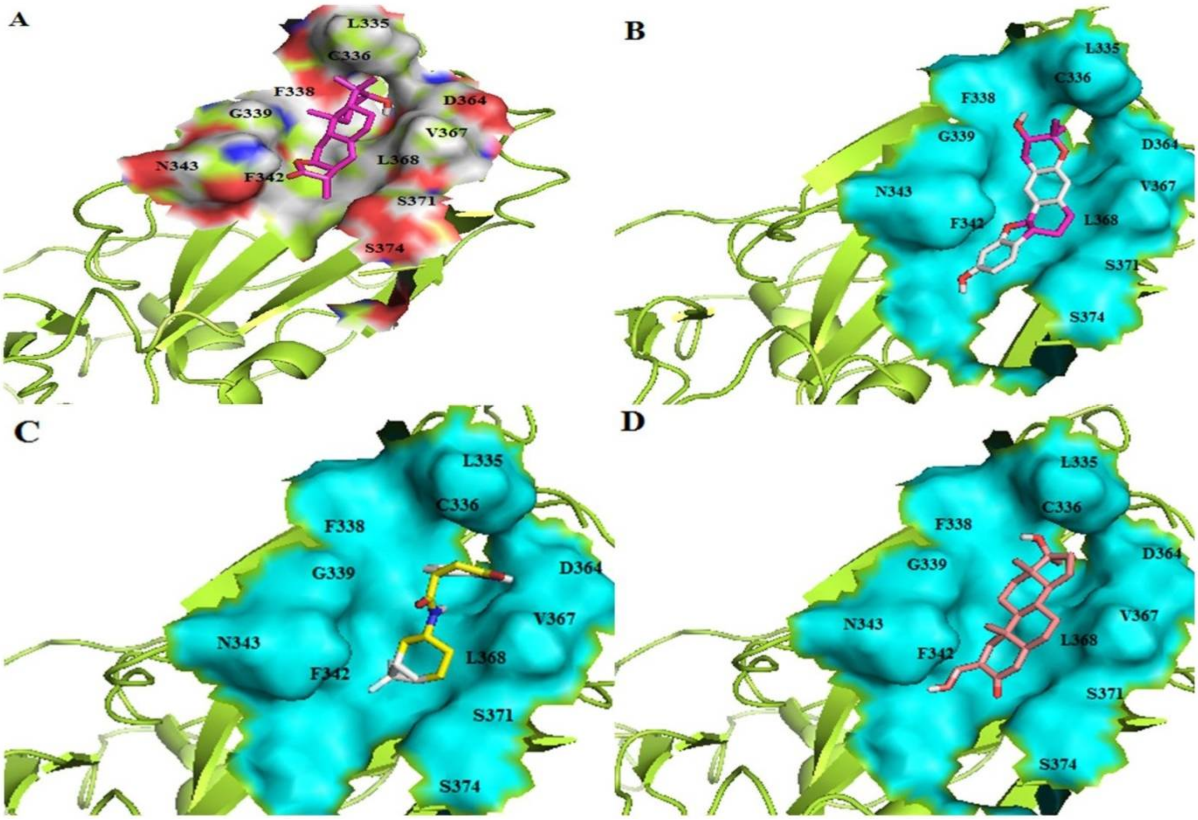

Figure S1: Cartoon representation of RBD in complex with: (a) NANPDB2245 (helioscopinolide B), (b) ZINC000095486008, (c) ZINC001645993538, and (d) Oxymetholone.

A

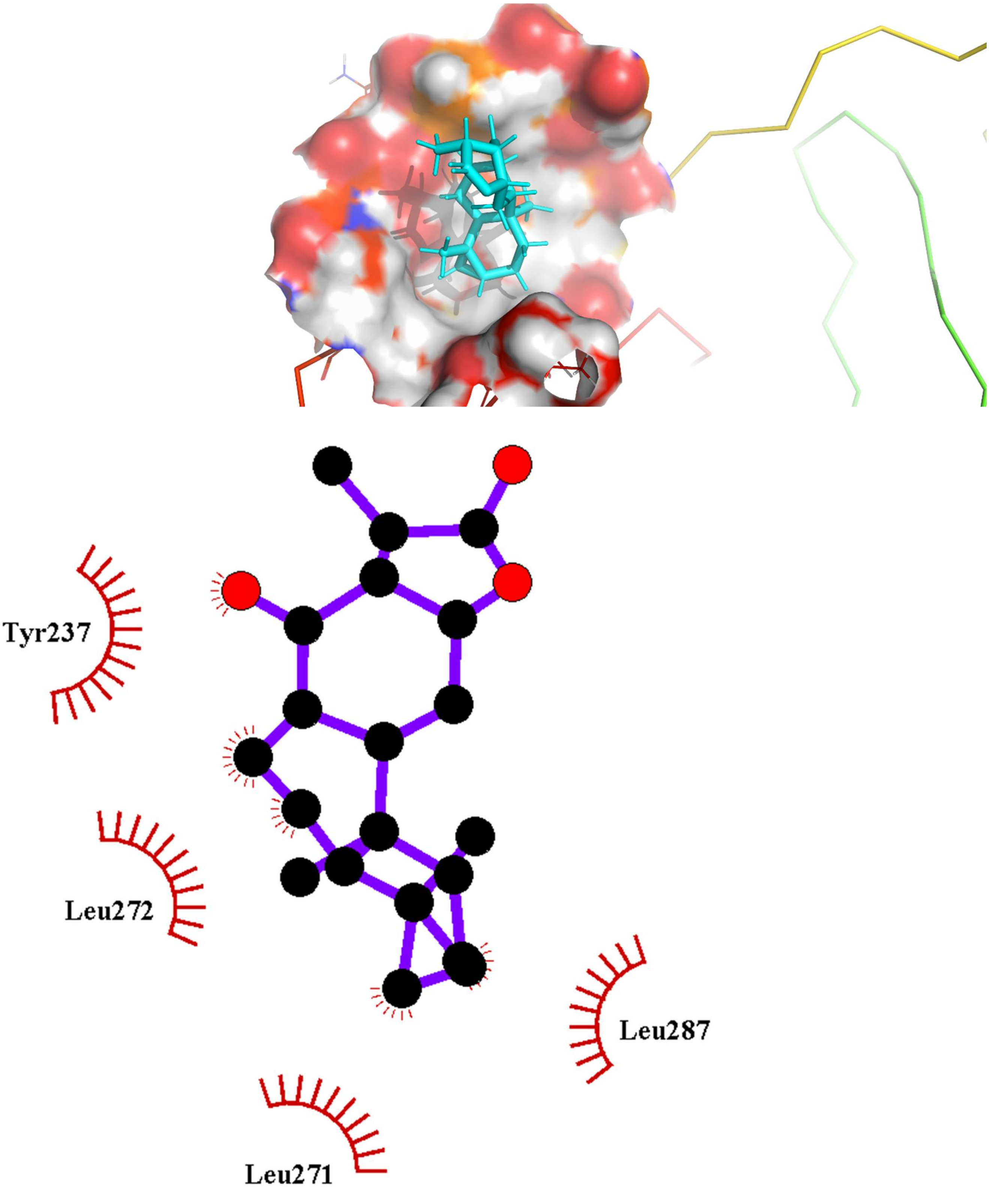

B

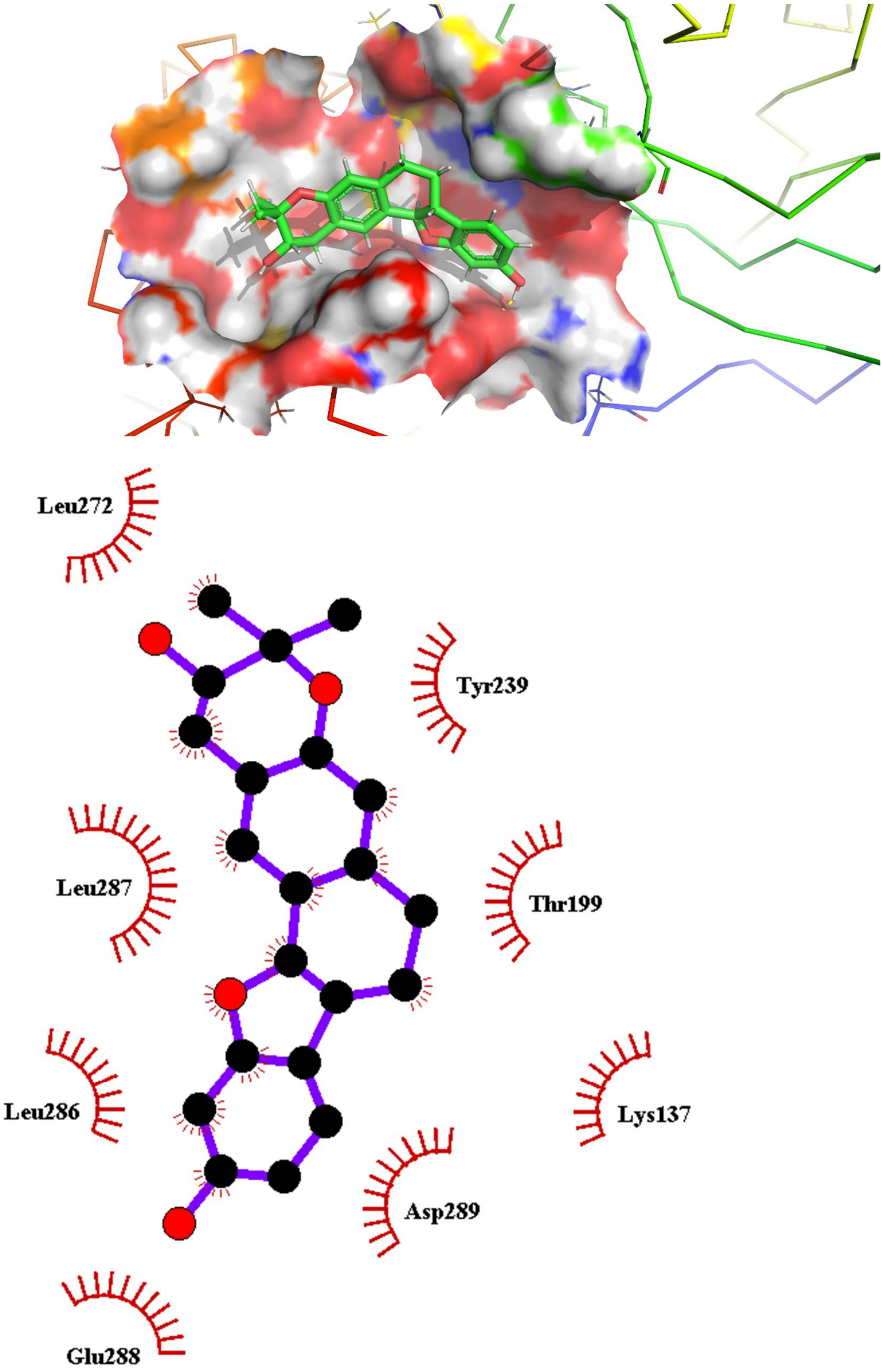

C

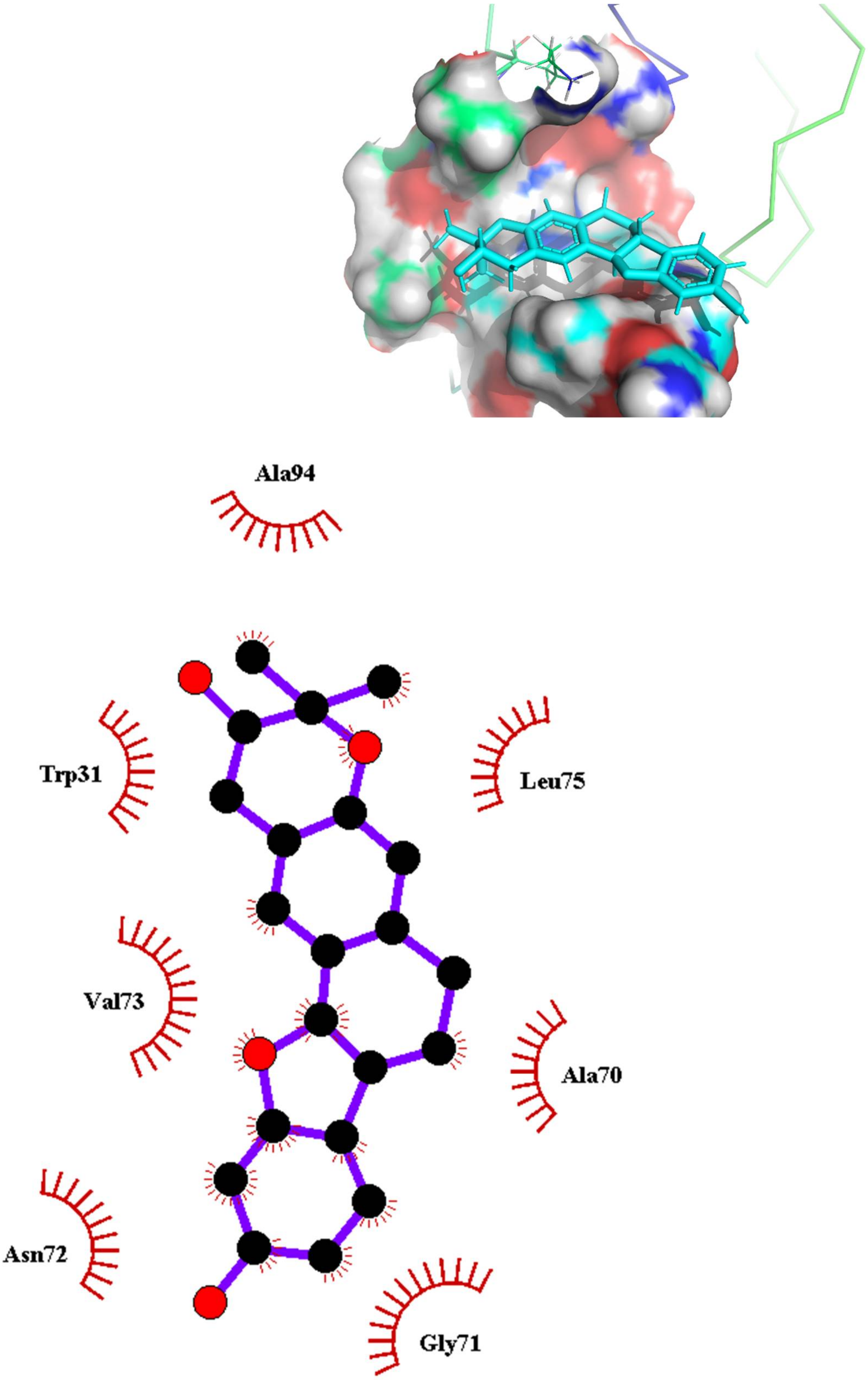

D

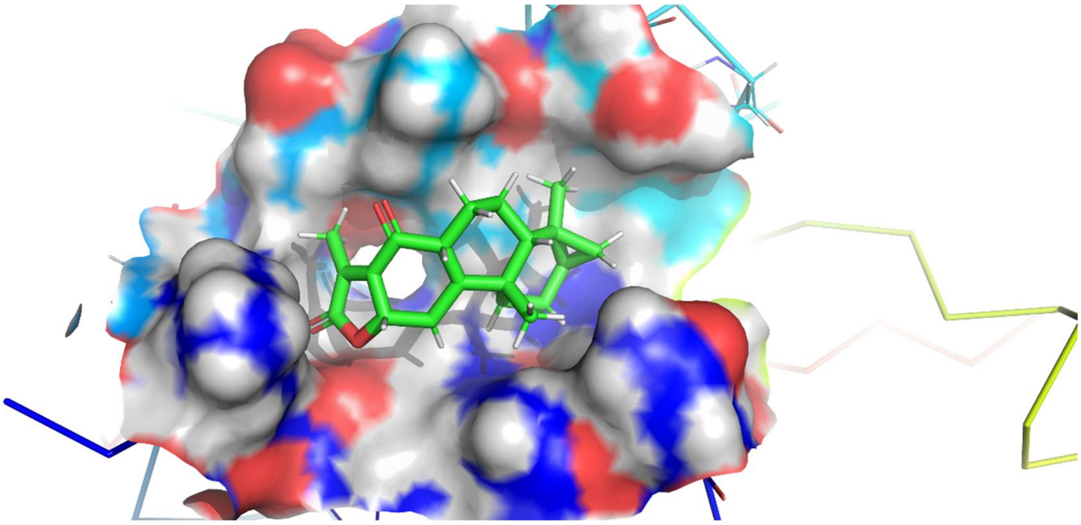

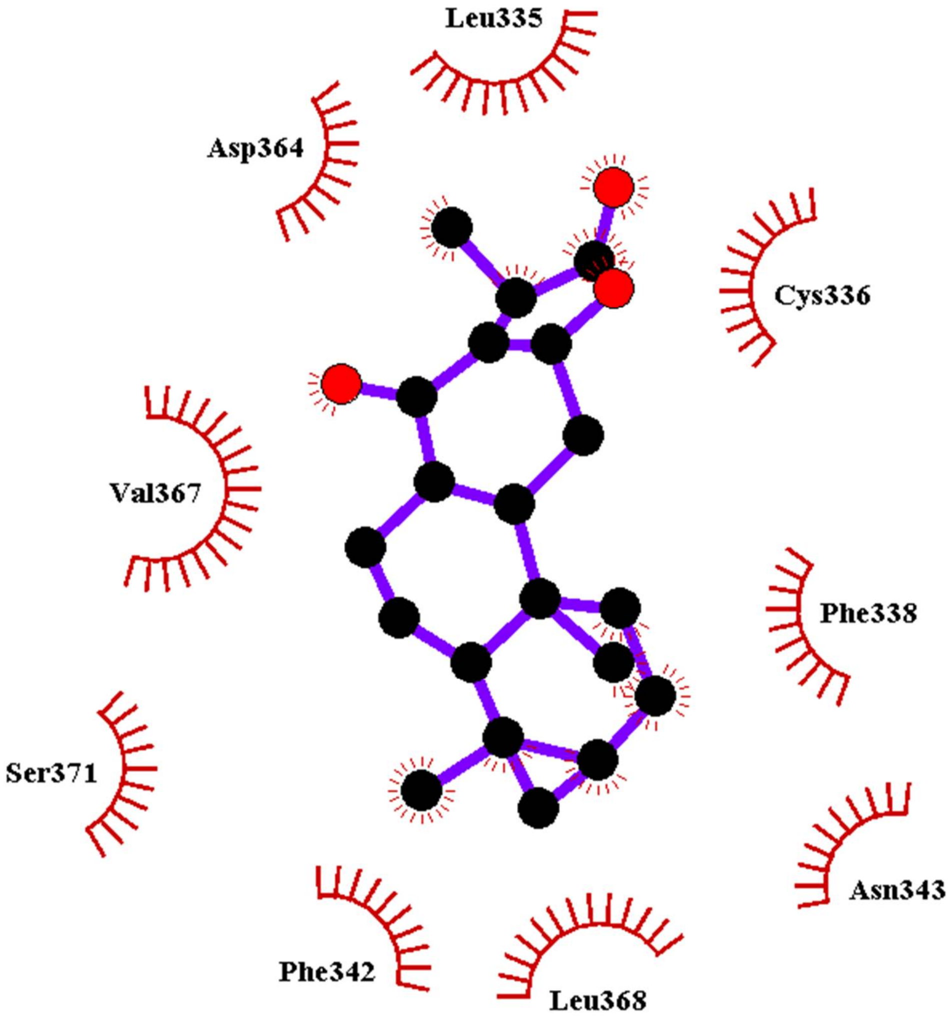
Leu335
Asp364
Cys336
Val367
Phe338
Ser371
Asn343
Phe342
Leu368

E

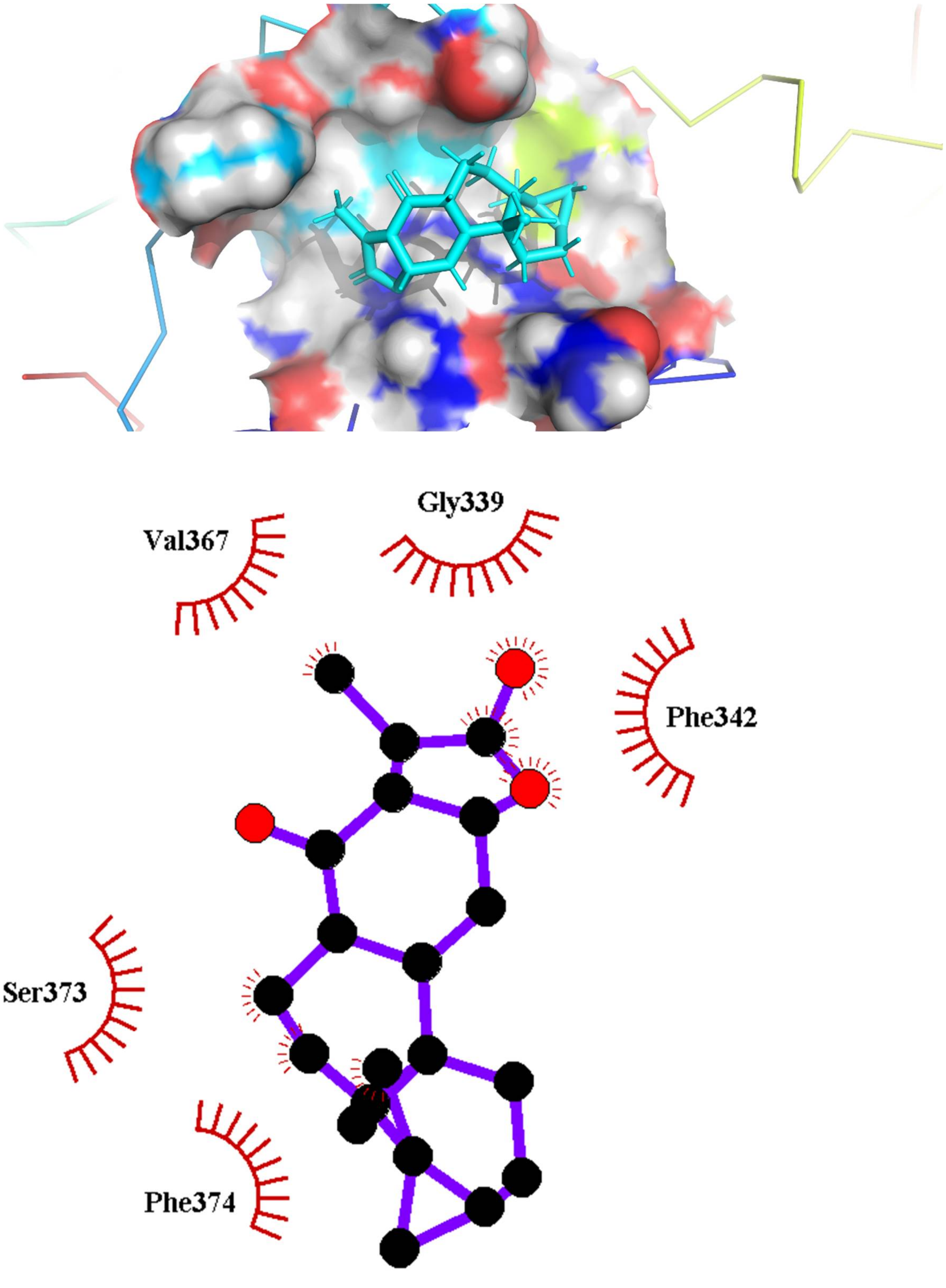

F

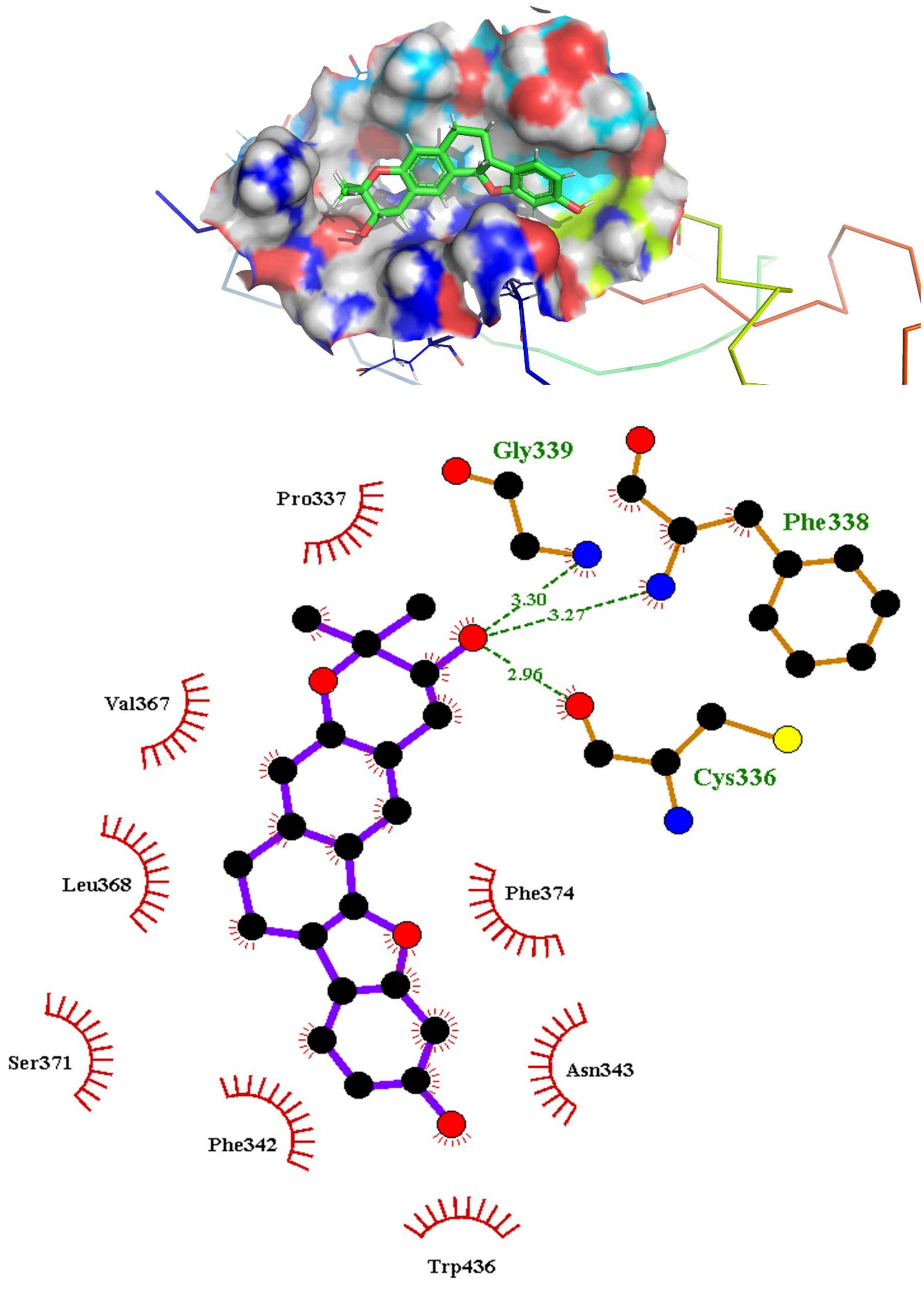
Gly339
Pro337
Phe338
3.30
3.27
2.96
Val367
Cys336
Leu368
Phe374
Ser371
Asn343
Phe342
Trp436

G

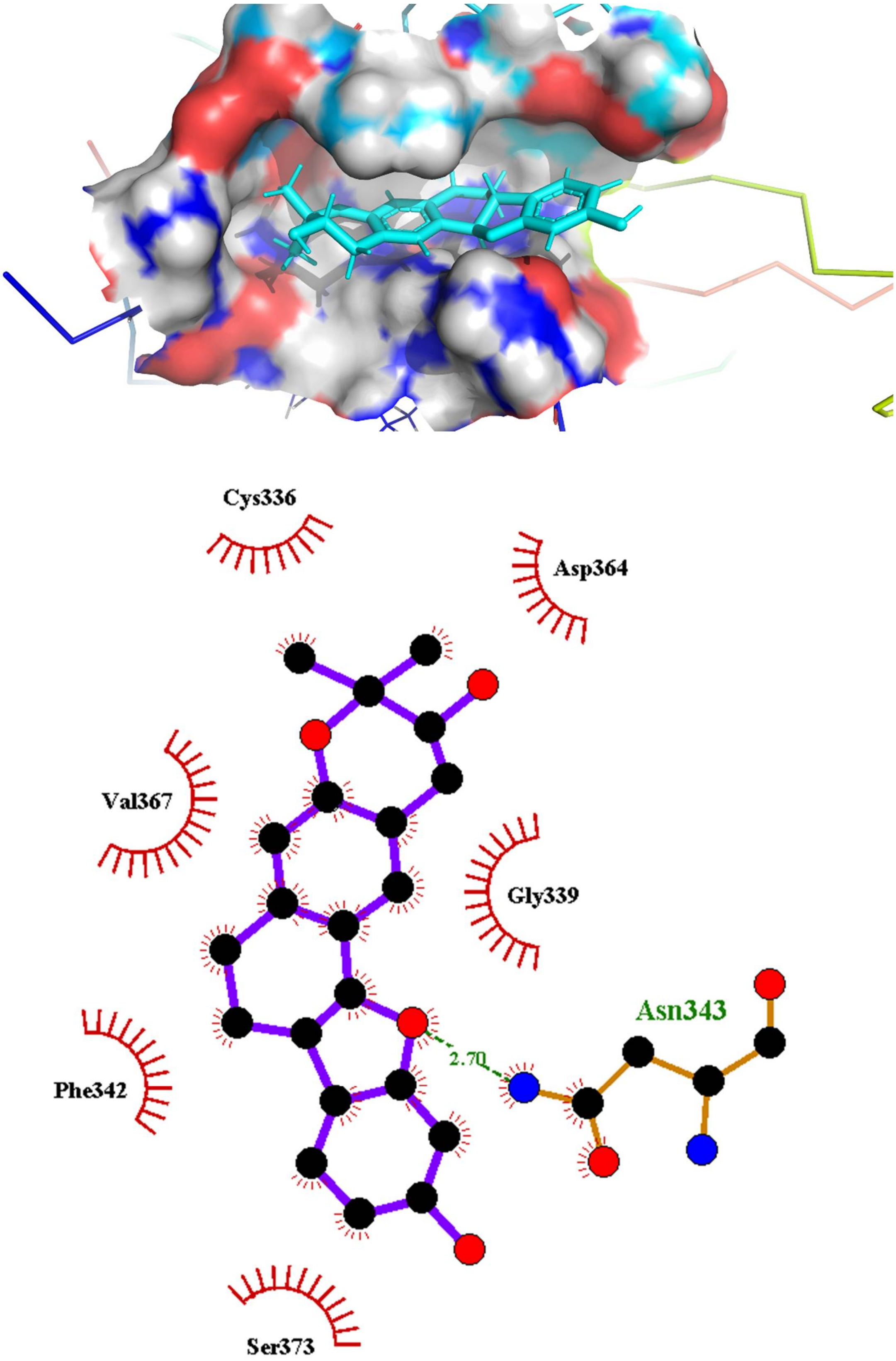

H

Figure S2: Binding mode representation and LigPlot+ characterization of: (a) M^{pro} and NANPDB2403 before MD simulation (pre-MD), (b) M^{pro} and NANPDB2403 after MD simulation (post-MD), (c) M^{pro} and ZINC95486008 before MD simulation (pre-MD), (d) M^{pro} and ZINC95486008 after MD simulation (post-MD), (e) RBD and NANPDB2403 before MD simulation (pre-MD), (f) RBD and NANPDB2403 after MD simulation (post-MD), (g) RBD and ZINC95486008 before MD simulation (pre-MD), and (h) RBD and ZINC95486008 after MD simulation (post-MD).

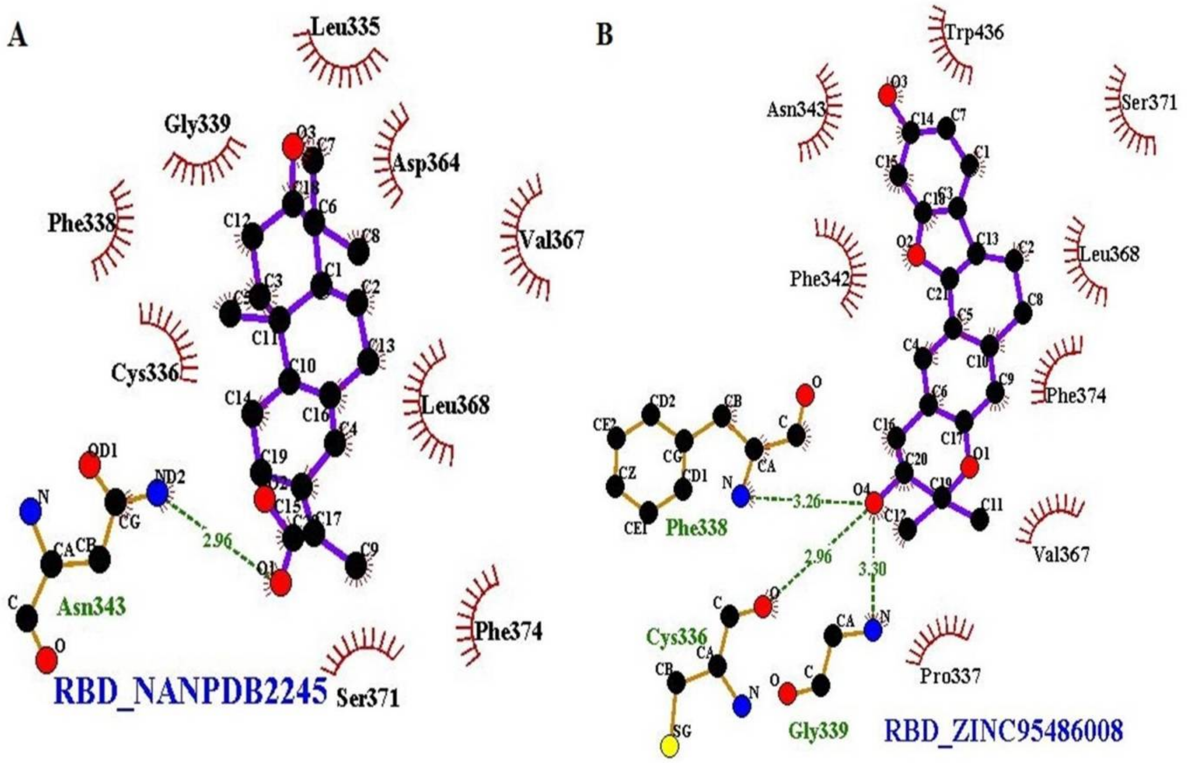

Figure S3: Two-dimensional diagram of the RBD–ligand interaction generated using LigPlot+. (a) Interaction profile of the RBD-NANPDB2245 complex, and (b) Interaction profile of the RBD-ZINC000095486008 complex.

Table S1: The binding energies and intermolecular interactions between the hits and M^{pro} as well as RBD.

COMPOUND	SOURCE	BINDING ENERGY (Kcal/mol)		HYDROGEN BONDS [BOND LENGTH (Å)]		HYDROPHOBIC BONDS	
		M^{pro}	RBD	M^{pro}	RBD	M^{pro}	RBD
Ledipasvir		-9.6	-9.9	Met276 (2.92)	Gly339 (2.91)	Lys5, Gly124, Tyr126, Gln127, Lys137, Gly138, Ser139, Thr199, Tyr237, Tyr239, Leu272, Gly275,	Leu335, Cys336, Pro337, Phe338, Phe342, Asn343, Ala363, Asp364, Leu368, Ser371, Ala372, Ser373,

						Asn277, Gly278, Leu286, Leu287, Glu290	Phe374, Ser375, Trp436, Asn437, Tyr508
Velpatasvir		-8.9	-8.5	Lys137 (3.19), Tyr237 (3.08)	Thr345 (3.09), Asn354 (3.03)	Lys5, Val125, Tyr126, Gly138, Ser139, Asp197, Thr199, Asn238, Leu271, Leu272, Leu286, Leu287, Gln290	Glu340, Val341, Ala344, Arg346, Phe347, Ala348, Ser349, Lys356, Ser399, Tyr449, Asn450, Leu452, Phe490, Leu492, Ser494
Imatinib		-8.5	-8.1	Leu282 (2.86), Glu288 (3.18)	-	Lys5, Thr199, Trp207, Leu271, Ser284, Leu286, Leu287, Asp289, Glu290, Phe291	Leu335, Phe338, Gly339, Phe342, Asn343, Ala344, Asp364, Leu368, Ser371, Ser373, Phe374, Trp436, Leu441, Arg509
ZINC001657931232	ML	-8.4	-7.8	Asp289 (2.9)	Asp364 (2.95), Ser371 (3.0, 3.20)	Thr199, Leu271, Leu272, Gly275, Met276, Tyr239, Leu286, Leu287, Glu288	Leu335, Cys336, Phe338, Phe342, Asn343, Ala363, Val367, Leu368, Ser373, Phe374, Trp436
ZINC001181689720	ML	-8.3	-7.5	Lys137 (3.21)	-	Thr199, Tyr237, Tyr239, Leu271, Leu272, Leu286, Leu287, Glu288, Asp289	Leu335, Cys336, Phe338, Gly339, Phe342, Asn343, Val362, Ala363, Asp364, Leu368, Ser371, Ser373, Phe374
ZINC000095486008	AFRO	-8.2	-7.8	Lys5 (3.1), Glu288 (3.02)	Cys336 (2.96), Phe338 (3.26), Gly339 (3.3)	Lys137, Asp197, Thr199, Tyr239, Leu272, Leu286, Leu287, Asp289, Glu290	Pro337, Phe342, Asn343, Val367, Leu368, Ser371, Phe374, Trp436
ZINC001460974086	ML	-8.2	-7.6	Phe140 (3.21), Leu141 (2.91)	Ser399 (2.96)	Thr25, Thr26, His41, Ser46, Met49, Asn142, Cys145, His163, Met165, Glu166, His172	Glu340, Val341, Ala344, Arg346, Phe347, Ala348, Ser349, Ala352, Asn354, Lys356, Asn450
ZINC000035941652	AFRO	-8.1	-7.9	Lys137 (3.12), Glu288 (2.97), Glu290 (3.29)	Val341 (3.02), Ser399 (2.90)	Thr199, Tyr239, Leu271, Leu272, Leu286, Leu287, Asp289	Glu340, Ala344, Arg346, Phe347, Ala348, Ser349, Asn354, Asn450
ZINC000002004122	AFRO	-8.1	-7.6	Lys137 (2.97), Thr199 (3.00), Asp289 (3.08)	-	Arg131, Tyr239, Leu271, Leu272, Gly275, Leu286, Leu287,	Cys336, Phe342, Asn343, Val367, Leu368, Ser371, Ser373, Phe374, Trp436
NANPDB2403	AFRO	-8.1	-7.8	Leu287 (3.22)	-	Thr199, Tyr237, Tyr239, Leu271, Leu272, Leu286.	Leu335, Cys336, Phe338, Phe342, Asn343, Asp364, Val367, Leu368, Ser371,

NANPDB2586	AFRO	-8.1	-7.8	Glu166 (3.14)	-	Thr24, Thr25, Thr45, Ser46, Met49, Asn142, Leu141, Cys145, His163, Met165	Phe338, Phe342, Asn343, Val367, Leu368, Ser371, Ser373
ZINC001177833419	ML	-8.1	-7.6	Lys137 (3.21), Thr199 (2.8), Asp289 (2.93), Glu290 (3.26)	Phe515 (2.89), Leu517 (2.97)	Arg131, Tyr239, Leu271, Leu272, Leu286, Leu287	Val382, Leu390, Phe392, Asp428, Phe429, Thr430, Glu516
Dactinomycin		-8.1	-7.8	Lys137 (3.27), Asn238 (2.75)	Arg355 (3.05), Lys462 (2.9)	Gly138, Gly170, Val171, Thr196, Asp197, Thr199, Tyr237, Tyr239, Leu272, Leu286, Leu287	Arg357, Tyr396, Pro426, Asp428, Phe464, Glu465, Arg466
ZINC000031168265	AFRO	-8.0	-7.5	Leu287 (3.06, 3.15), Asp289 (3.11)	Arg355 (3.04)	Thr199, Tyr237, Leu271, Leu272, Leu286	Tyr396, Pro426, Asp428, Pro463, Phe464, Glu516
NANPDB2245	AFRO	-8.0	-7.7	Arg131 (2.92)	Asn343 (2.96)	Lys137, Thr199, Tyr237, Tyr239, Leu271, Leu272, Leu286, Leu287, Asp289	Leu335, Cyc336, Phe338, Gly339, Asp364, Val367, Leu368, Ser371, Phe374
ZINC000055656943	ML	-8.0	-8.0	Asp197 (2.80)	-	Arg131, Thr198, Thr199, Tyr237, Tyr239, Leu272, Leu287	Leu335, Cys336, Phe338, Phe342, Asp364, Val367, Leu368, Ser371, Phe374,
ZINC000636416501	ML	-8.0	-7.9	-	Ser494 (3.1), Asn501 (3.19)	Val104, Gln110, Asn151, Asp153, Ser158, Phe294, Arg298	Arg403, Tyr495, Gly496, Gln498, Gly502, Tyr505
Dolutegravir		-8.0	-8.0	Thr199 (3.14), Leu287 (2.84)	Ser399 (2.95)	Lys137, Tyr237, Tyr239, Leu272, Asp289	Glu340, Ala344, Arg346, Phe347, Ala348, Ser349, Tyr351, Ala352, Asn354, Asn450, Leu452
ZINC000000134782	AFRO	-7.9	-7.9	Arg298 (3.12)	-	Val104, Ile106, Gln110, Thr111, Asn151, Ser158, Thr292, Phe294	Phe338, Phe342, Asn343, Val367, Leu368, Ser371, Ser373, Phe374, Trp436
NANPDB2510	AFRO	-7.9	-7.6	Arg131 (2.93)	Asn343 (2.9)	Lys137, Thr199, Tyr237, Leu271, Leu272, Leu286, Leu287, Asp289	Leu335, Cys336, Phe338, Gly339, Val367, Leu368, Ser371, Phe374
ZINC000014557836	AFRO	-7.9	-7.6	Thr199 (2.79), Leu271 (2.98), Leu287 (2.7), Asp289 (2.82, 3.21)	Asn501 (2.83, 3.30)	Leu272, Gly275, Leu286, Glu288, Glu290	Arg403, Tyr453, Ser494, Tyr495, Gly496, Gly502, Tyr505
ZINC001337920081	ML	-7.9	-8.1	Glu14 (3.21), Gln69 (2.96), Ser121 (3.01)	Arg346 (2.93),	Gly15, Gln19, Trp31, Ala70, Gly71, Lys97, Asn119, Gly120, Pro122	Glu340, Val341, Ala344, Phe347, Ala348, Ser349, Ala352, Asn354, Ser399

					Asn450 (3.1, 3.24)		
ZINC001240794368	ML	-7.9	-7.8	Asp289 (3.16)	Thr430 (3.03)	Arg131, Asp197, Thr198, Thr199, Tyr237, Leu271, Leu272, Leu286, Leu287	Pro426, Phe429, Pro463, Phe464, Phe515, Glu516
Bictegravir		-7.9	-7.9	Lys5 (2.92), Lys137 (2.8), Glu288 (2.83)	Phe342 (3.22)	Tyr126, Gln127, Arg131, Gly138, Leu286, Leu287, Glu290	Phe338, Gly339, Asn343, Leu368, Ser371, Ser373, Phe374, Trp436, Asn437, Asn440
Oxymetholone		-7.8	-7.7	Thr25 (2.81), Glu166 (2.9, 3.00)	Cys336 (3.0), Asn343 (3.09)	His41, Ser46, Thr45, Asn142, Gly143, Cys145, His164, Met165	Leu335, Phe338, Gly339, Phe342, Asp364, Val367, Leu368, Ser371, Phe374
Raltegravir		-7.8	-7.5	Lys5 (3.08), Lys137 (3.03), Glu290 (3.04)	Asn501 (2.82), Tyr505 (3.17)	Tyr126, Cys128, Arg131, Gly138, Thr199, Tyr239, Leu286, Leu287, Glu288, Asp289	Tyr449, Gln493, Ser494, Tyr495, Gly496, Gly502
Sirolimus		-7.8	-7.9	ND	ND	ND	ND
ZINC000095485910	AFRO	-7.7	-7.9	Lys137 (2.93)	Gly496 (2.89, 2.91), Asn501 (3.09)	Thr199, Tyr237, Tyr239, Leu286, Leu287, Asp289	Arg403, Glu406, Lys417, Tyr453, Tyr495, Phe497, Tyr505
ZINC001645993538	ML	-7.7	-7.5	Thr199 (314)	-	Lys137, Asp197, Tyr239, Leu272, Leu286, Leu287, Glu288, Asp289	Cys336, Phe338, Asp364, Val367, Leu368, Ser371, Phe374
ZINC000373659060	ML	-7.7	-7.5	Thr25 (3.07), His164 (2.81)	Thr430 (3.21), Phe515 (3.09)	His41, Thr45, Ser46, Met49, Asn142, Cys145, Met165, Glu166, Gln189	Pro426, Phe429, Phe464, Ser514, Glu516, Leu517,
ZINC000621286015	ML	-7.7	-7.6	-	Ser349 (2.96, 3.14), Asn354 (3.01), Ser399 (3.07), Asn450 (3.04, 3.16)	Glu14, Gly15, Met17, Val18, Trp31, Ala70, Gly71, Lys97, Asn119, Gly120, Ser121	Val341, Arg346, Phe347, Ala348, Ala352
ZINC000043069427	AFRO	-7.6	-7.5	Asp197 (3.05), Asp289 (2.95)	Cys336 (2.89), Phe338 (2.97), Gly339 (3.13), Asp364 (2.79, 3.11)	Arg131., Thr198, Thr199, Tyr237, Tyr239, Leu271, Leu272, Leu286, Leu287	Phe342, Asn343, Ala363, Val367, Leu368, Ser373, Phe374, Trp436
ZINC001198818678	ML	-7.6	-7.5	Leu141 (2.92), Ser144 (3.2), Glu166 (2.97)	Ser371 (2.97)	Thr25, Thr45, Ser46, Met49, Phe140, Asn142, His163, Met165	Cys336, Phe338, Gly339, Phe342, Asn343, Asp364, Val367, Leu368, Ser373, Phe374, Trp436

ZINC000542648459	ML	-7.6	-7.6	Leu287 (3.30)	Cys336 (2.96), Phe338 (3.16), Gly339 (2.9)	Thr199, Tyr237, Leu272, Leu286, Asp289	Phe342, Asn343, Asp364, Val367, Leu368, Phe374
NANPDB2412	AFRO	-7.5	-7.7	Arg131 (3.14)	Phe338 (3.27)	Lys137, Thr199, Tyr237, Tyr239, Leu272, Leu286, Leu287, Asp289	Leu335, Cys336, Gly339, Phe342, Val367, Leu368, Ser371, Phe374
ZINC000544552417	ML	-7.5	-7.5	-	Phe338 (3.1), Gly339 (2.8)	Arg131, Lys137, Thr199, Tyr239, Leu272, Met276, Leu286, Leu287, Glu288, Asp289	Leu335, Cys336, Pro337, Phe342, Asn343, Asp364, Val367, Leu368, Ser371, Phe374,
Fusidic acid		-6.9	-7.2	Lys137 (2.8), Leu271 (2.94), Leu272 (3.05)	Ser371 (2.7), Ser373 (2.92)	Arg131, Asp197, Thr199, Tyr239, Gly275, Met276, Leu286, Asp289	Leu335, Cys336, Gly339, Phe342, Asn343, Val367, Leu368, Phe374, Trp436

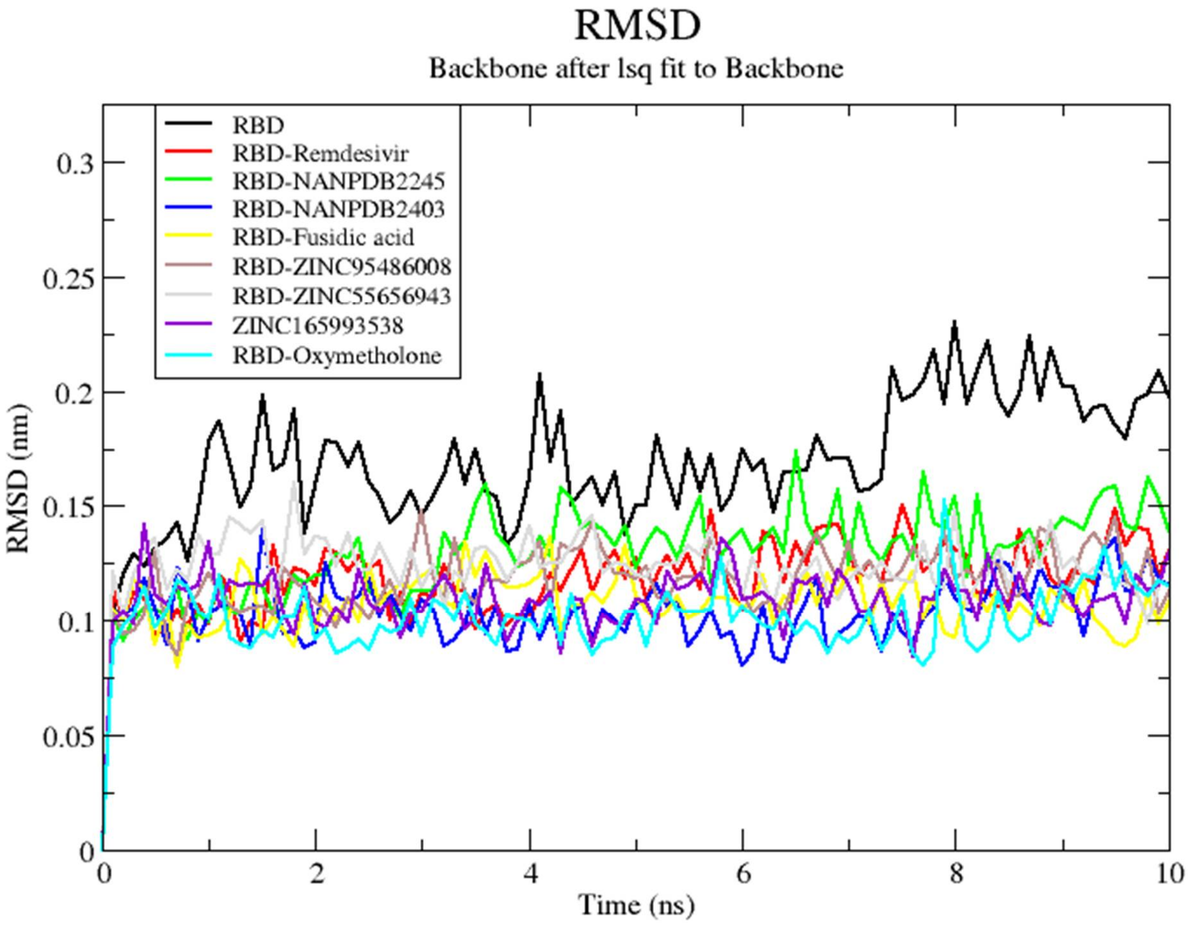

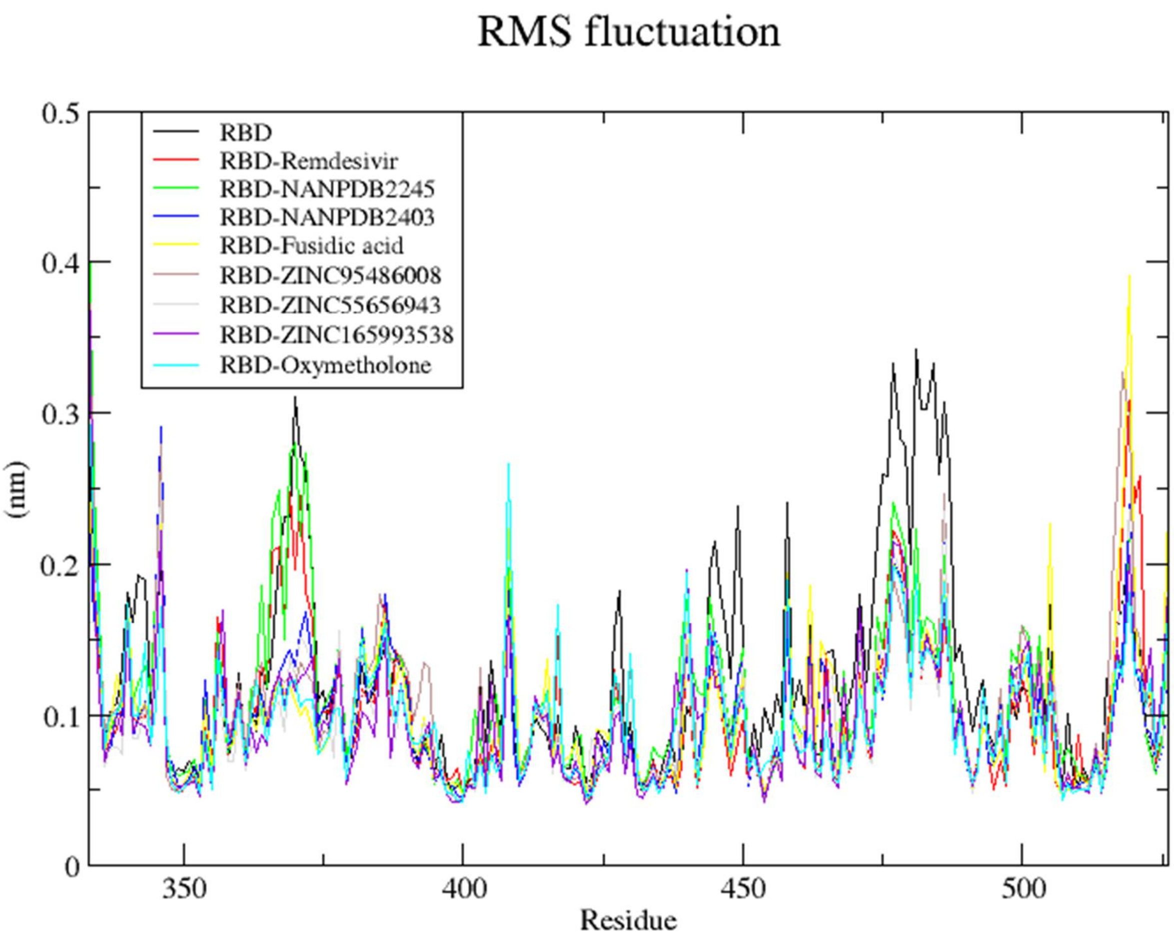

RMS fluctuation
RBD
RBD-Remdesivir
RBD-NANPDB2245
RBD-NANPDB2403
RBD-Fusidic acid
RBD-ZINC95486008
RBD-ZINC55656943
RBD-ZINC165993538
RBD-Oxymetholone
(nm)
Residue
0
0.1
0.2
0.3
0.4
0.5
350
400
450
500

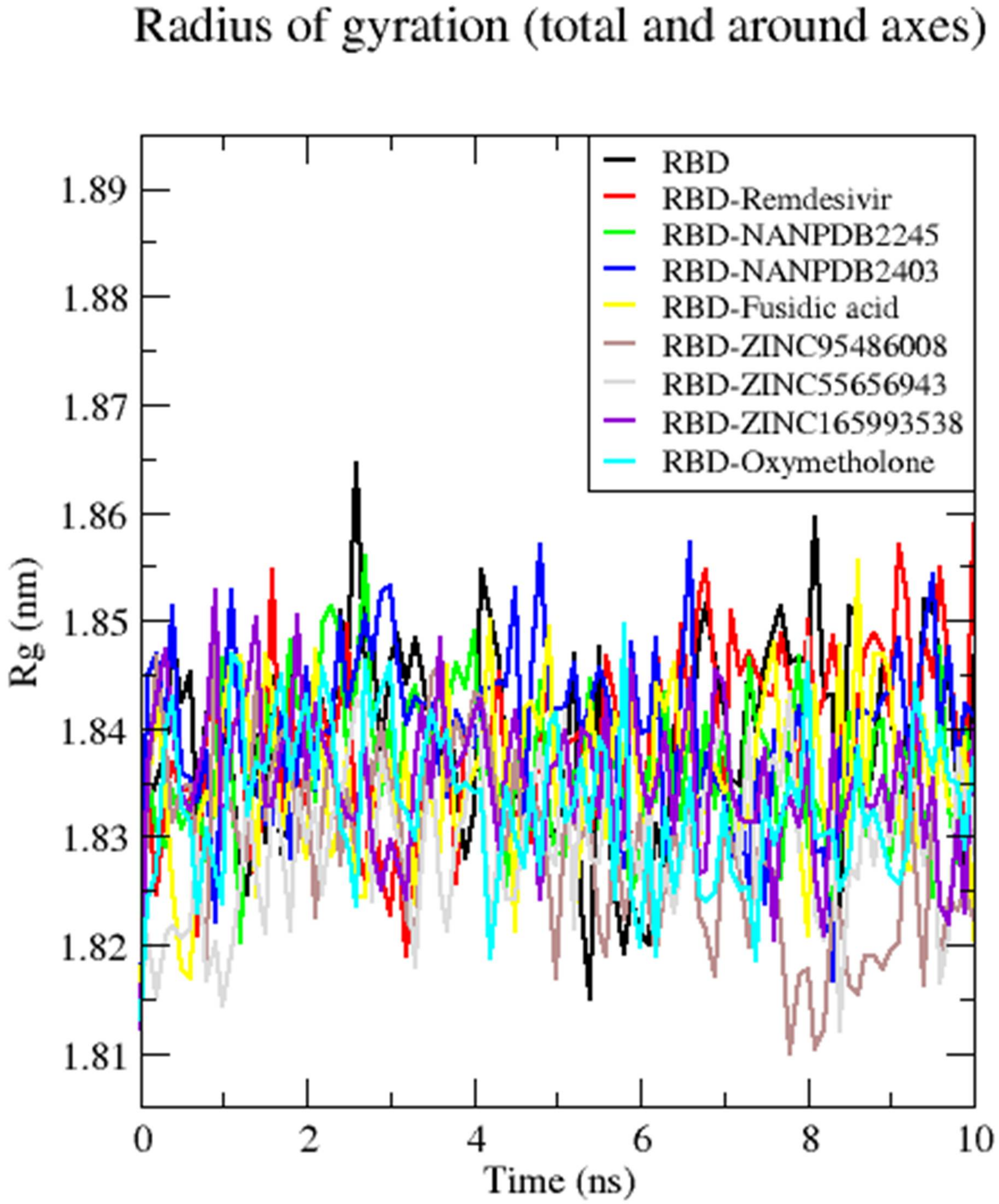

Figure S4: Graphs of the RMSD, RMSF and radius of gyration of the RBD–ligand complexes generated over a 10 ns molecular dynamics simulation using GROMACS. (a) RMSD versus time graph of the RBD–ligand complexes, (b) Analysis of the RMSF trajectories of the residues of the RBD–ligand complexes, and (c) Rg versus time graph of the RBD–ligand complexes.

A) M^{pro}-Remdesivir

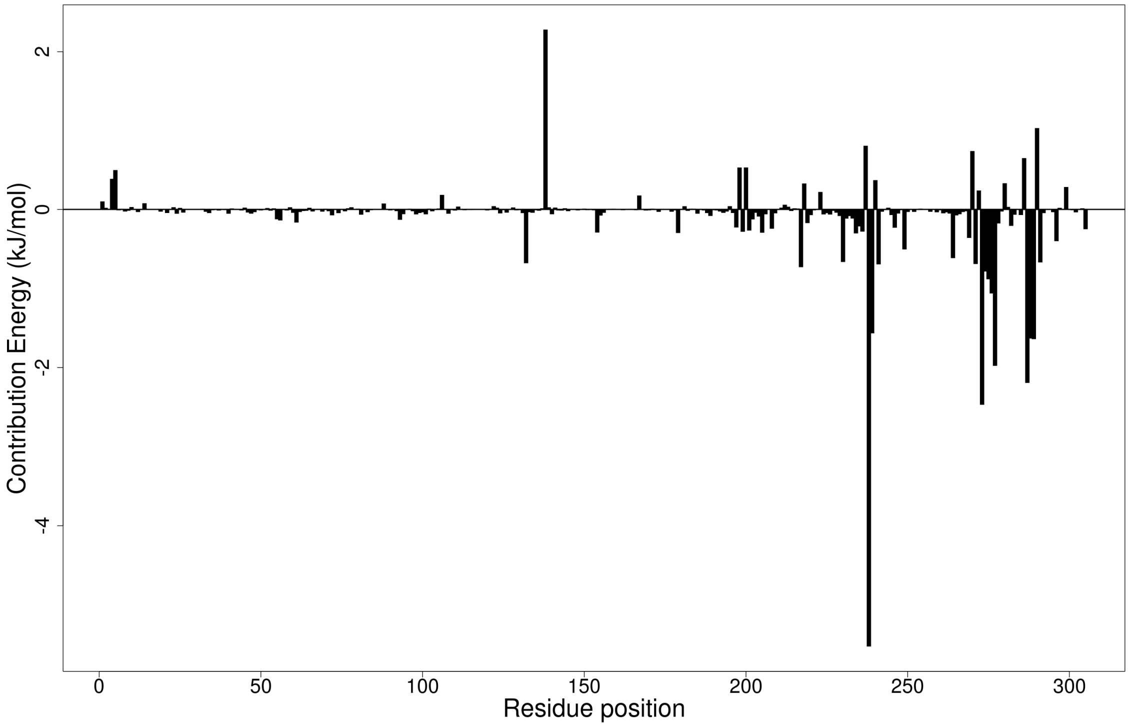

B) M^{pro}-NANPDB2245

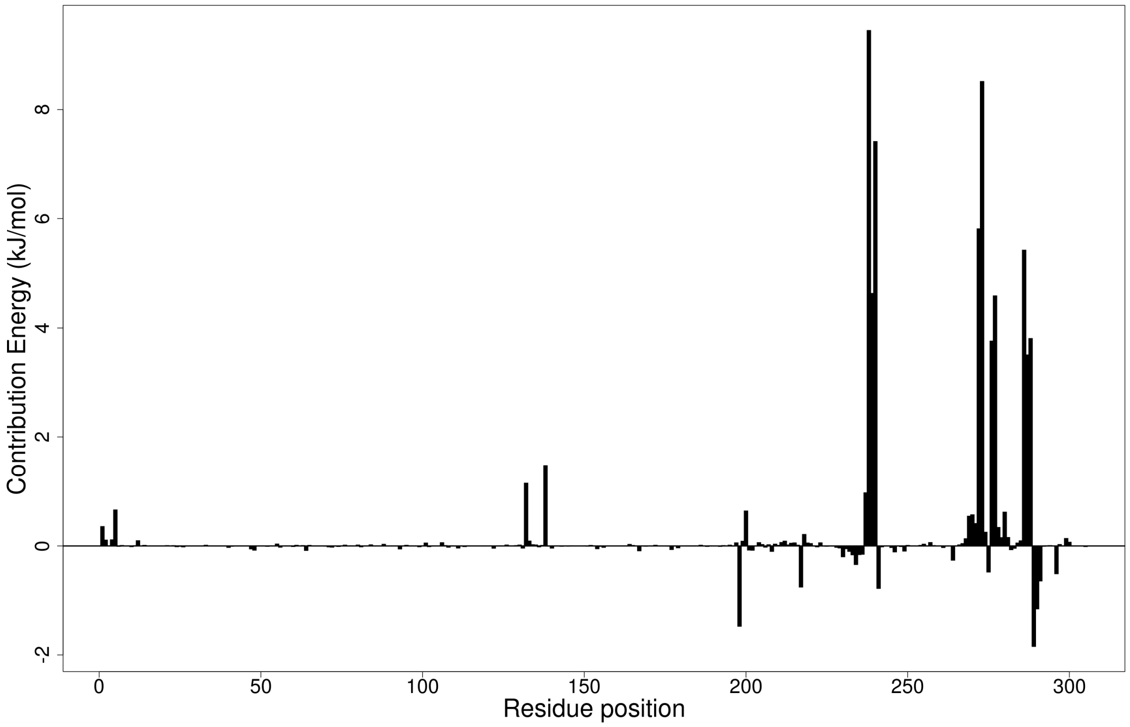

C) M^{pro}-NANPDB2403

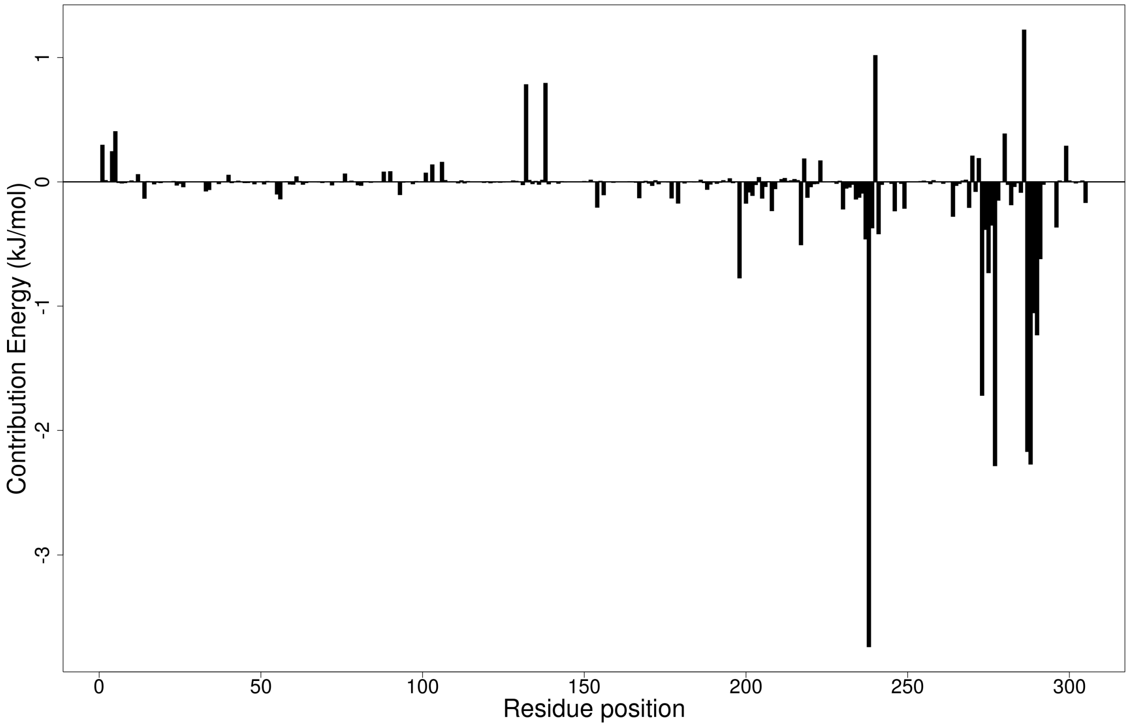

D) M^{pro}-Fusidic acid

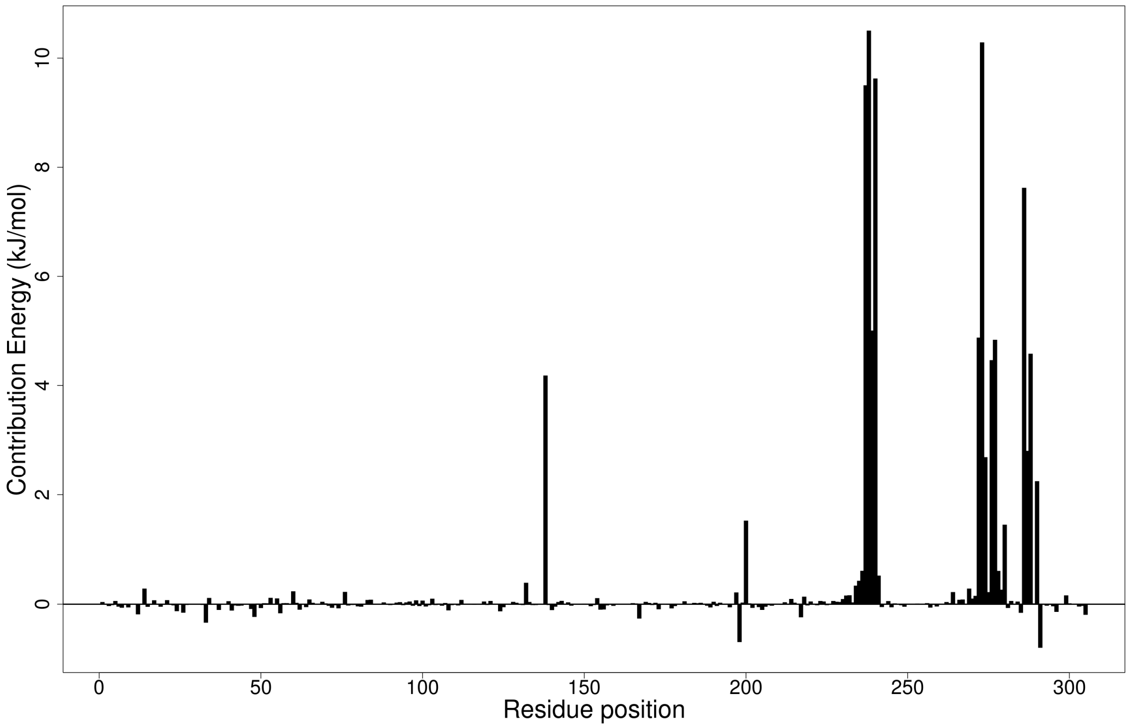

E) M^{pro}-ZINC000055656943

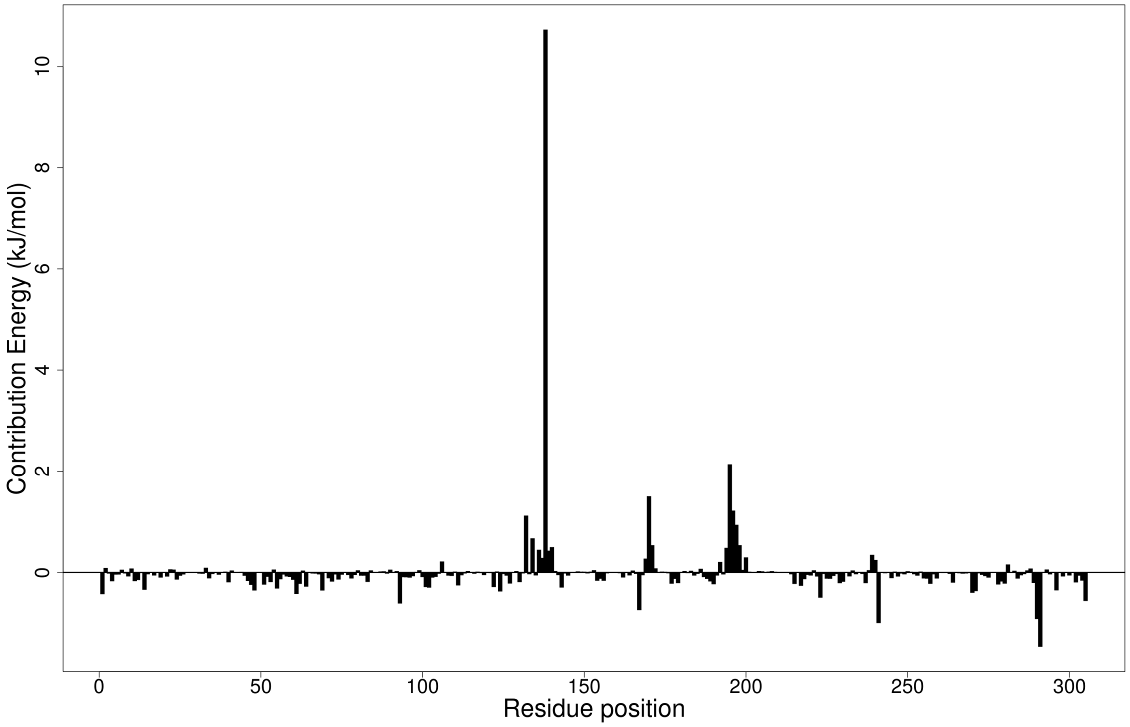

F) M^{pro}-ZINC001645993538

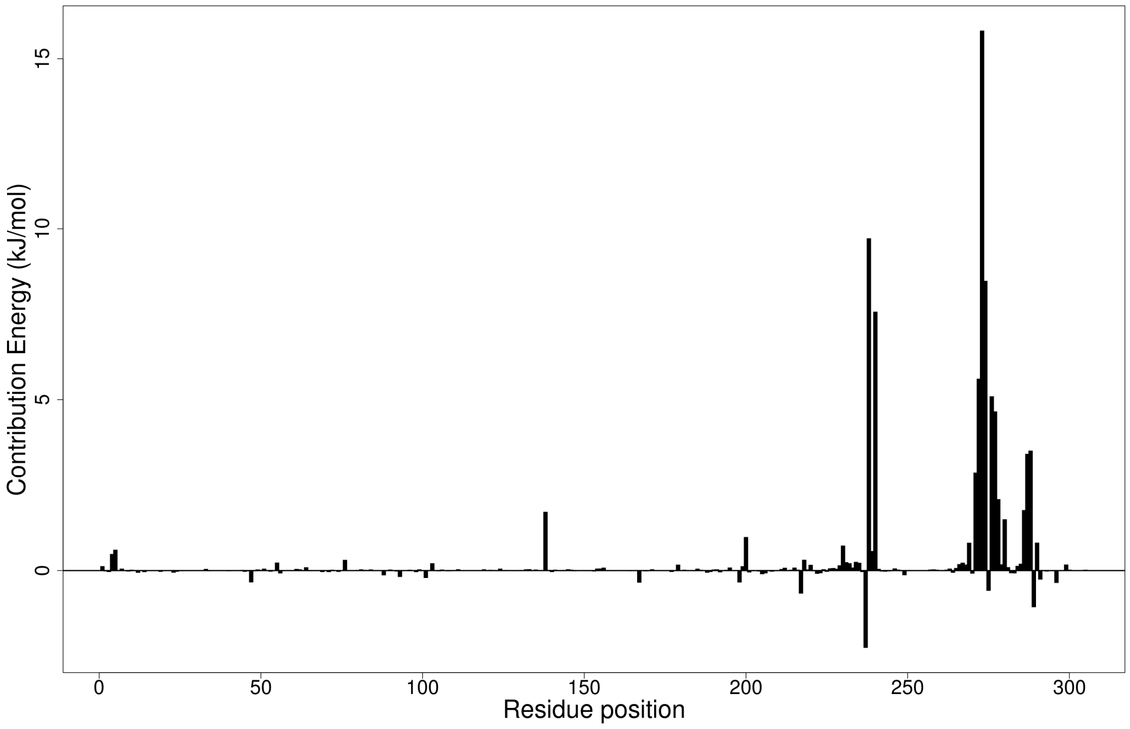

G) M^{pro}-Oxymetholone

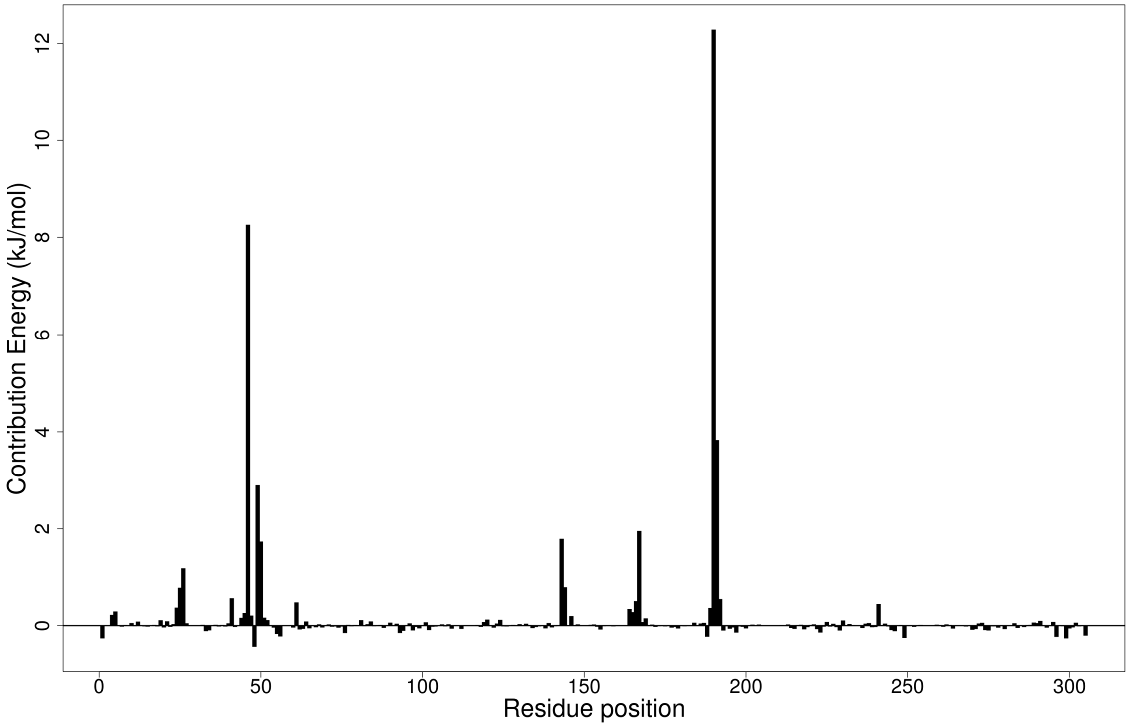

H) RBD-Remdesivir

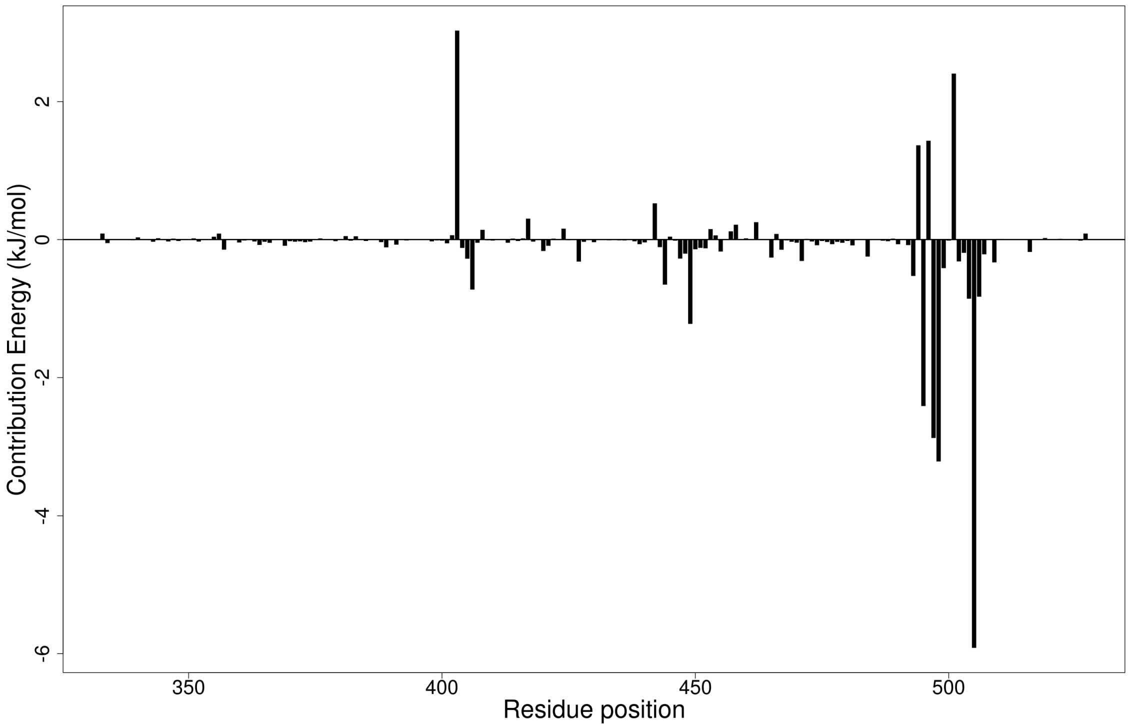

I) RBD-NANPDB2245

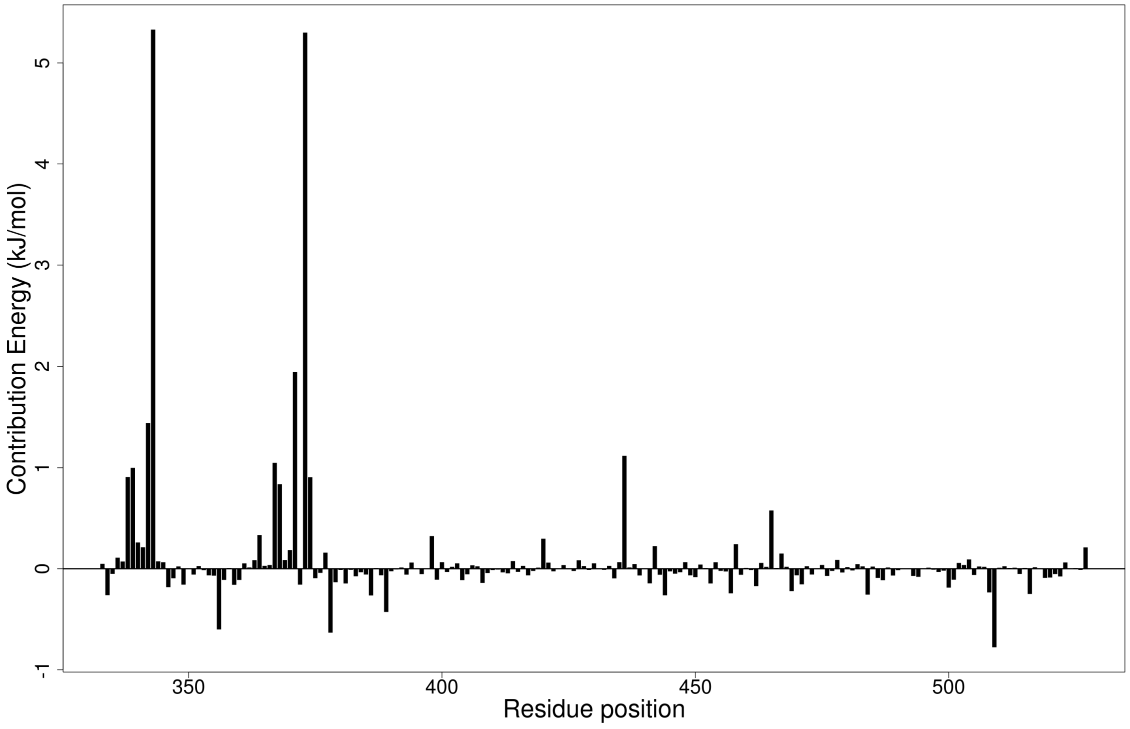

J) RBD-NANPDB2403

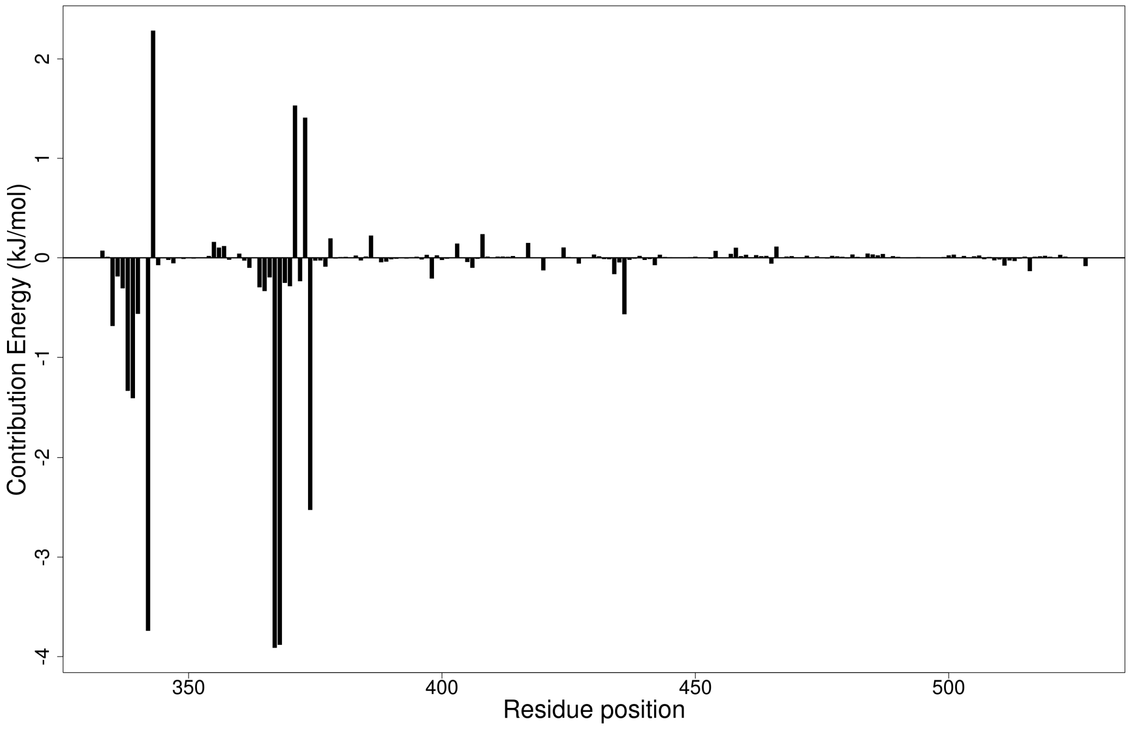

K) RBD-Fusidic acid

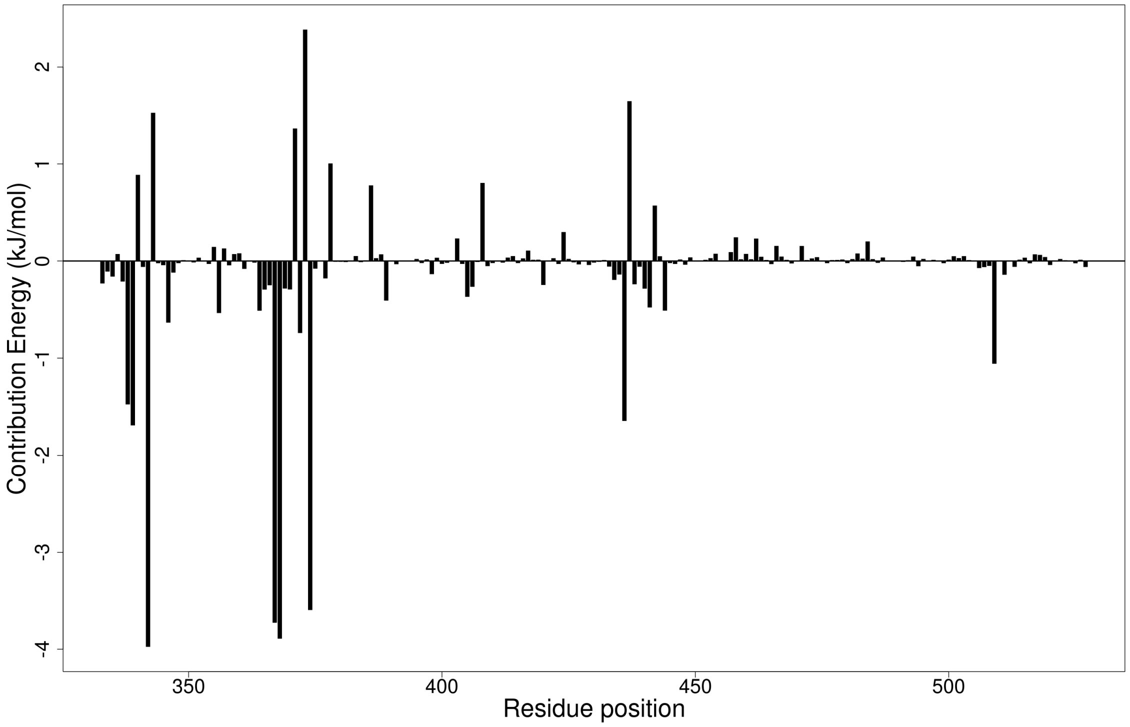

L) RBD-ZINC000095486008

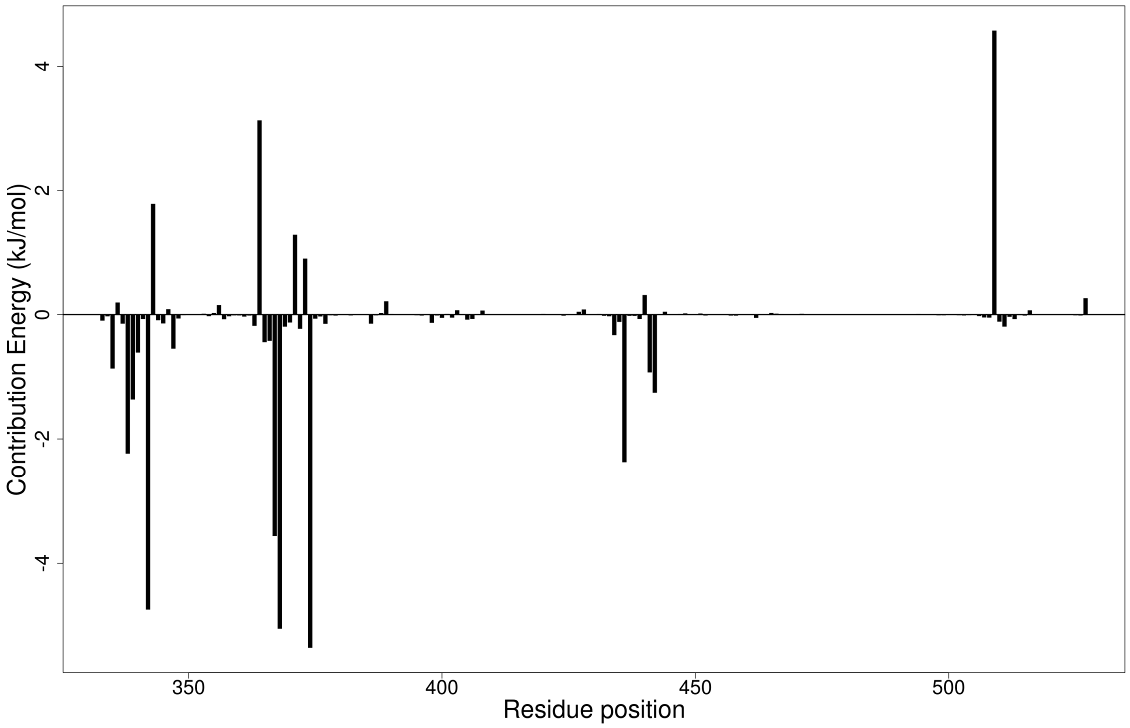

M) RBD- ZINC000055656943

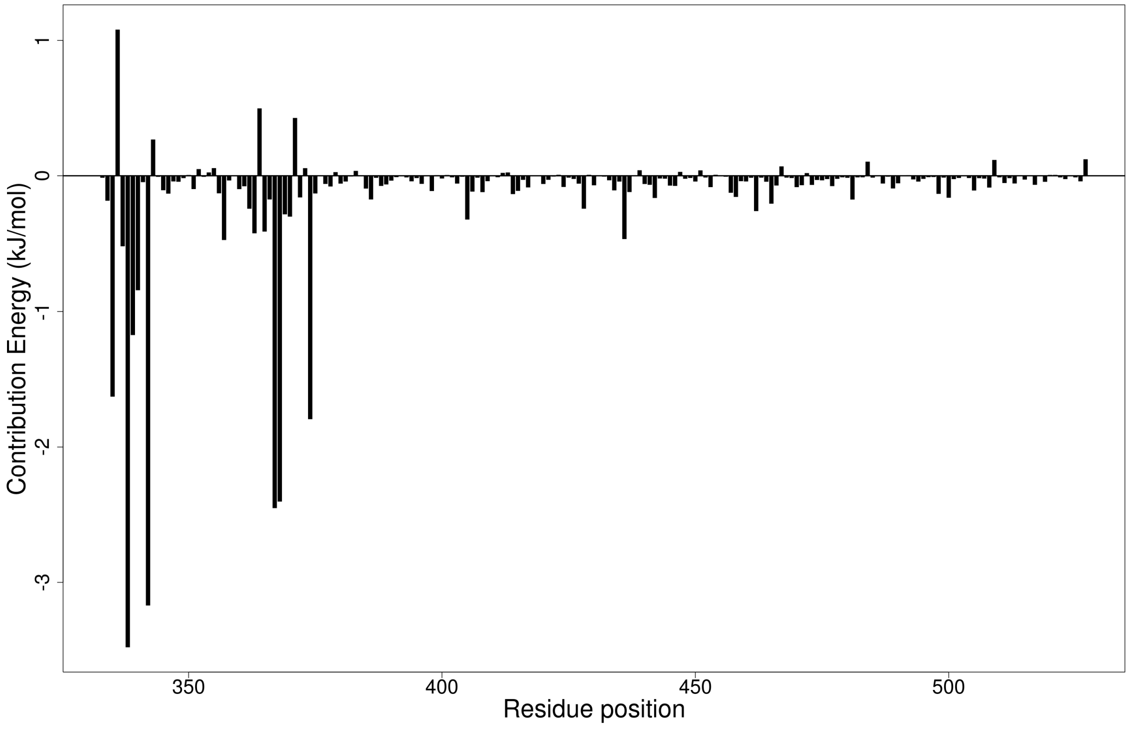

N) RBD- ZINC001645993538

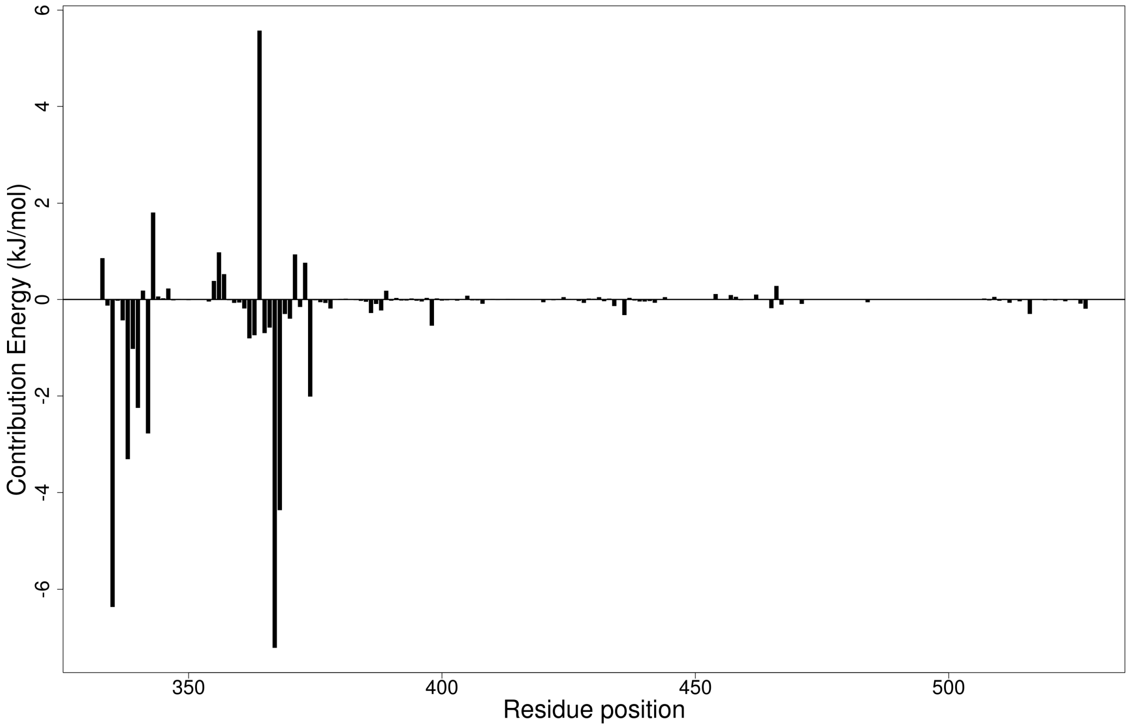

O) RBD-Oxymetholone

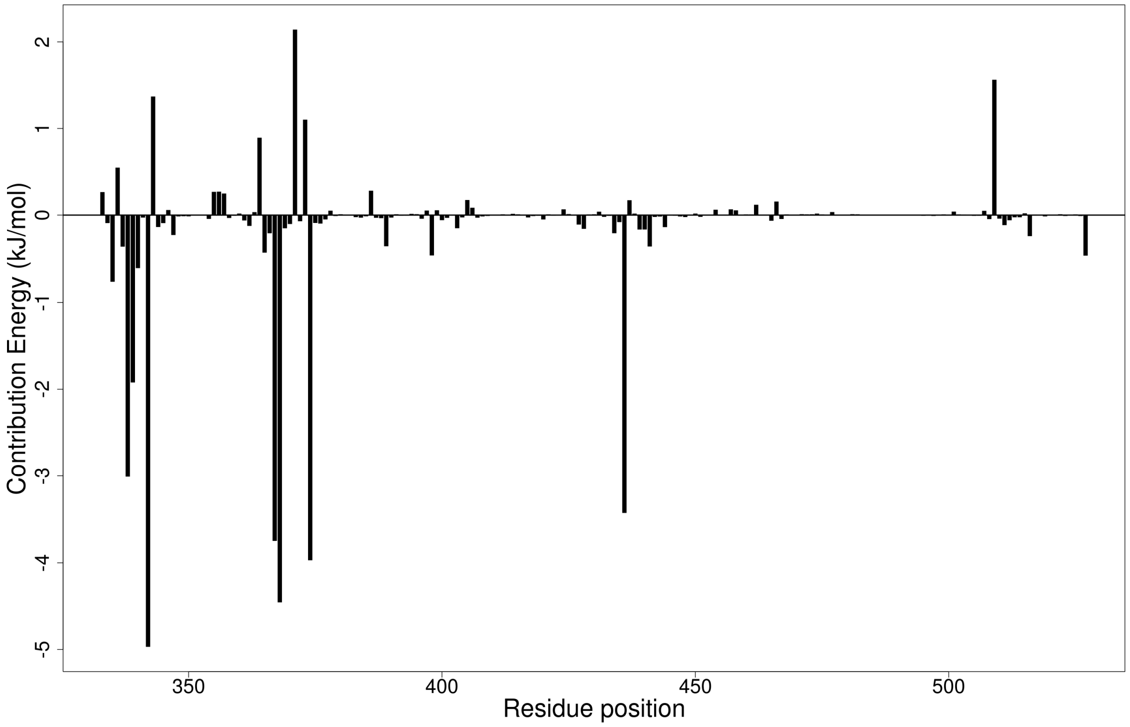

Figure S5: MM/PBSA plot of the binding free energy contribution per residue of the protein–ligand complexes (a) M^{pro}-Remdesivir, (b) M^{pro}-NANPDB2245, (c) M^{pro}-NANPDB2403, (d) M^{pro}-fusidic acid, (e) M^{pro}-ZINC000055656943, (f) M^{pro}-ZINC001645993538, (g) M^{pro}-oxymetholone, (h) RBD-Remdesivir, (i) RBD-NANPDB2245, (j) RBD-NANPDB2403, (k) RBD-fusidic acid, (l) RBD-ZINC000095486008, (m) RBD-ZINC000055656943, (n) RBD-ZINC001645993538, and (o) RBD-Oxymetholone.
